# Overlaying human and mosquito behavioral data to estimate residual exposure to host-seeking mosquitoes and the protection of bednets in a malaria elimination setting where indoor residual spraying and nets were deployed together

**DOI:** 10.1371/journal.pone.0270882

**Published:** 2022-09-15

**Authors:** Lucia Fernandez Montoya, Celso Alafo, Helena Martí-Soler, Mara Máquina, Kiba Comiche, Inocencia Cuamba, Khatia Munguambe, Lauren Cator, Pedro Aide, Beatriz Galatas, Nelson Cuamba, Dulcisaria Marrenjo, Francisco Saúte, Krijn P. Paaijmans

**Affiliations:** 1 ISGlobal, Hospital Clínic—Universitat de Barcelona, Barcelona, Spain; 2 Centro de Investigação em Saúde de Manhiça (CISM), Fundação Manhiça, Mozambique; 3 Goodbye Malaria, Tchau Tchau Malaria Foundation, Chibungo, Mozambique; 4 Imperial College London, London, United Kingdom; 5 Instituto Nacional da Saúde, Ministério da Saúde, Maputo, Mozambique; 6 Programa Nacional de Controlo da Malária, Ministério da Saúde, Maputo, Mozambique; 7 PMI VectorLink Project, Abt Associates Inc., Maputo, Mozambique; 8 Center for Evolution and Medicine, School of Life Sciences, Arizona State University, Tempe, AZ, United States of America; 9 The Biodesign Center for Immunotherapy, Vaccines and Virotherapy, Arizona State University, Tempe, AZ, United States of America; 10 Simon A. Levin Mathematical, Computational and Modeling Sciences Center, Arizona State University, Tempe, AZ, United States of America; Swiss Tropical and Public Health Institute, SWITZERLAND

## Abstract

Characterizing persistent malaria transmission that occurs after the combined deployment of indoor residual spraying (IRS) and long-lasting insecticidal nets (LLINs) is critical to guide malaria control and elimination efforts. This requires a detailed understanding of both human and vector behaviors at the same temporal and spatial scale. Cross-sectional human behavior evaluations and mosquito collections were performed in parallel in Magude district, Mozambique. Net use and the exact time when participant moved into each of five environments (outdoor, indoor before bed, indoor in bed, indoor after getting up, and outdoor after getting up) were recorded for individuals from three different age groups and both sexes during a dry and a rainy season. Malaria mosquitoes were collected with CDC light traps in combination with collection bottle rotators. The percentage of residual exposure to host-seeking vectors that occurred in each environment was calculated for five local malaria vectors with different biting behaviors, and the actual (at observed levels of LLIN use) and potential (i.e. if all residents had used an LLIN) personal protection conferred by LLINs was estimated. *Anopheles arabiensis* was responsible for more than 74% of residents’ residual exposure to host-seeking vectors during the Magude project. The other four vector species (*An*. *funestus s*.*s*., *An*. *parensis*, *An*. *squamosus* and *An*. *merus)* were responsible for less than 10% each. The personal protection conferred by LLINs prevented only 39.2% of the exposure to host-seeking vectors that survived the implementation of both IRS and LLINs, and it differed significantly across seasons, vector species and age groups. At the observed levels of bednet use, 12.5% of all residual exposure to host-seeking vectors occurred outdoor during the evening, 21.9% indoor before going to bed, almost two thirds (64%) while people were in bed, 1.4% indoors after getting up and 0.2% outdoor after leaving the house. Almost a third of the residual exposure to host-seeking vectors (32.4%) occurred during the low transmission season. The residual bites of *An*. *funestus s*.*s*. and *An*. *parensis* outdoors and indoor before bedtime, of *An*. *arabiensis* indoors when people are in bed, and of *An*. *squamosus* both indoors and outdoors, are likely to have sustained malaria transmission throughout the Magude project. By increasing LLIN use, an additional 24.1% of exposure to the remaining hosts-seeking vectors could have been prevented. Since *An*. *arabiensis*, the most abundant vector, feeds primarily while people are in bed, increasing net use and net feeding inhibition (through e.g. community awareness activities and the selection of more effective LLINs) could significantly reduce the exposure to remaining host-seeking mosquitoes. Nonetheless, supplementary interventions aiming to reduce human-vector contact outdoors and/or indoors before people go to bed (e.g. through larval source management, window and eave screening, eave tubes, and spatial repellents) will be needed to reduce residual exposure to the outdoor and early biting *An*. *funestus s*.*s*. and *An*. *parensis*.

## Introduction

Mozambique is one of the four countries with the highest malaria burden in the world [1]. Reducing and eventually eliminating malaria in its most southern province (Maputo province) has been considered critical to make progress towards malaria elimination in South Africa and Eswatini as well. Although Maputo province has been targeted by regional initiatives aiming at accelerating malaria elimination, such as LSDI (Lubombo Spatial Development Initiative) [2] and MOSASWA (Mozambique, South Africa and Eswatini) [3], neither of these initiatives -nor previous attempts to eliminate malaria in sub-Saharan Africa- have succeeded in interrupting transmission. There is an urgent need to improve our understanding of the limitations of current control interventions in order to optimize them and/or implement novel or supplementary interventions [2,4], if we are to achieve malaria elimination in sub-Saharan Africa.

Malaria control has historically relied heavily on controlling malaria vectors through indoor residual spraying (IRS). Although IRS led to great reductions in the malaria burden in Africa during the Global Malaria Eradication Programme (GMEP) in the 1950s and 1960s, it was not sufficient to interrupt malaria transmission in Africa. It was concluded that IRS failed due to rapidly evolving insecticide resistance and the fact that some mosquito species were not resting indoors [5–8]. Since 2000, and due to renewed efforts to eliminate malaria, insecticide treated nets (ITNs), which were later replaced by long-lasting insecticidal nets (LLINs), have become the most widely used vector control intervention. ITNs, and to a lesser extent IRS, have contributed most to the observed reductions in malaria cases in Africa between 2000 and 2015 [9]. Challenges for LLINs include resistance to pyrethroids [10], the main insecticide class used in nets, and mosquitoes biting people when they are not under the net (either outdoors or indoors) [11].

IRS and LLINs target different mosquito behaviors. IRS reduces the survival of mosquitoes that rest on treated wall surfaces and, hence, vector population densities. LLINs protect people by killing mosquitoes, repelling them when they approach the net and by acting as a physical barrier, preventing vector-host contact. As pyrethroid resistance is widespread in Africa, the combination of IRS with a non-pyrethroid insecticide and LLINs (which are currently pyrethroid-based) could have an additional impact on malaria transmission, compared to implementing a single intervention [12], and can help to mitigate for the effects of insecticide resistance [13]. Such combinations could therefore play a critical role in accelerating malaria elimination in low transmission settings. However the scientific evidence of the added value of combining IRS with LLINs is limited and not always in agreement [14,15], which lead the WHO to call for additional evidence in malaria transmission foci, including low transmission settings [13].

Besides evaluating the added epidemiological value of combining the two interventions, we need to understand their gap(s) in protection, which was evaluated during the Magude project [16]. The project assessed the feasibly of eliminating malaria in a low transmission setting in southern Mozambique using a package of interventions targeting the malaria parasites and vectors simultaneously. Vector control consisted of the implementation of annual district-wide IRS in addition to LLINs that are mass-distributed every three years. Although the project achieved significant reductions in malaria incidence and prevalence, malaria transmission was not interrupted [17]. Hence, this project provides a unique opportunity to understand the gaps in protection (i.e. persistent interactions between humans and mosquitoes) that remain in a low malaria transmission setting after the combined deployment of the two core vector control interventions. To-date, such evaluations have focused on comparing the impact of the individual versus combined interventions on standard entomological indicators through mathematical models [18], through empirical data from experimental hut trials that mimic semi-field conditions [19] or through field studies [12,20–22]. But to accurately characterize residual malaria transmission, both human and vector behavioral data are needed to identify the place and time where and when humans and malaria vector species interact [13]. Although methods to quantify human exposure to mosquito bites were already developed in 2006 [23], very few studies have since collected empirical data to evaluate these human-vector interactions [24,25], and even fewer studies have collected human and mosquito behavioral data at the same time and in the same place [26–31]. In addition, no study has evaluated human-vector interactions in a low transmission setting where LLINs are combined with area-wide IRS.

Here, using both human and vector behavioral data that were collected in parallel in Magude between 2015 and 2017, we 1) estimate the proportion of residual exposure to five host-seeking vector species (i.e. mosquito species that survived the combined deployment of LLIN and IRS and were found carrying sporozoites) experienced by residents of Magude in each of five different environment: outdoors before going indoors, indoors before going to bed, indoors while in bed, indoors after getting up and outdoors after leaving the house; 2) assess the actual personal protection that LLINs conferred to Magude residents against the five different local malaria vector species; 3) estimate the maximum personal protection that LLINs could have conferred if all residents would have used a net to sleep; and 4) characterize the residual exposure to host-seeking mosquitoes that would have remained in each environment even if all residents would have used a net to sleep every night. To our knowledge, this is the first study to characterize the residual exposure to bites of different vector species (five) with distinct host-seeking patterns in an area with combined deployment of LLINs and IRS, and to report the protective efficacy of LLINs against those different vector species during both the low and high malaria transmission season.

## Materials and methods

### Study site and status of vector control interventions

**Fig 1:**
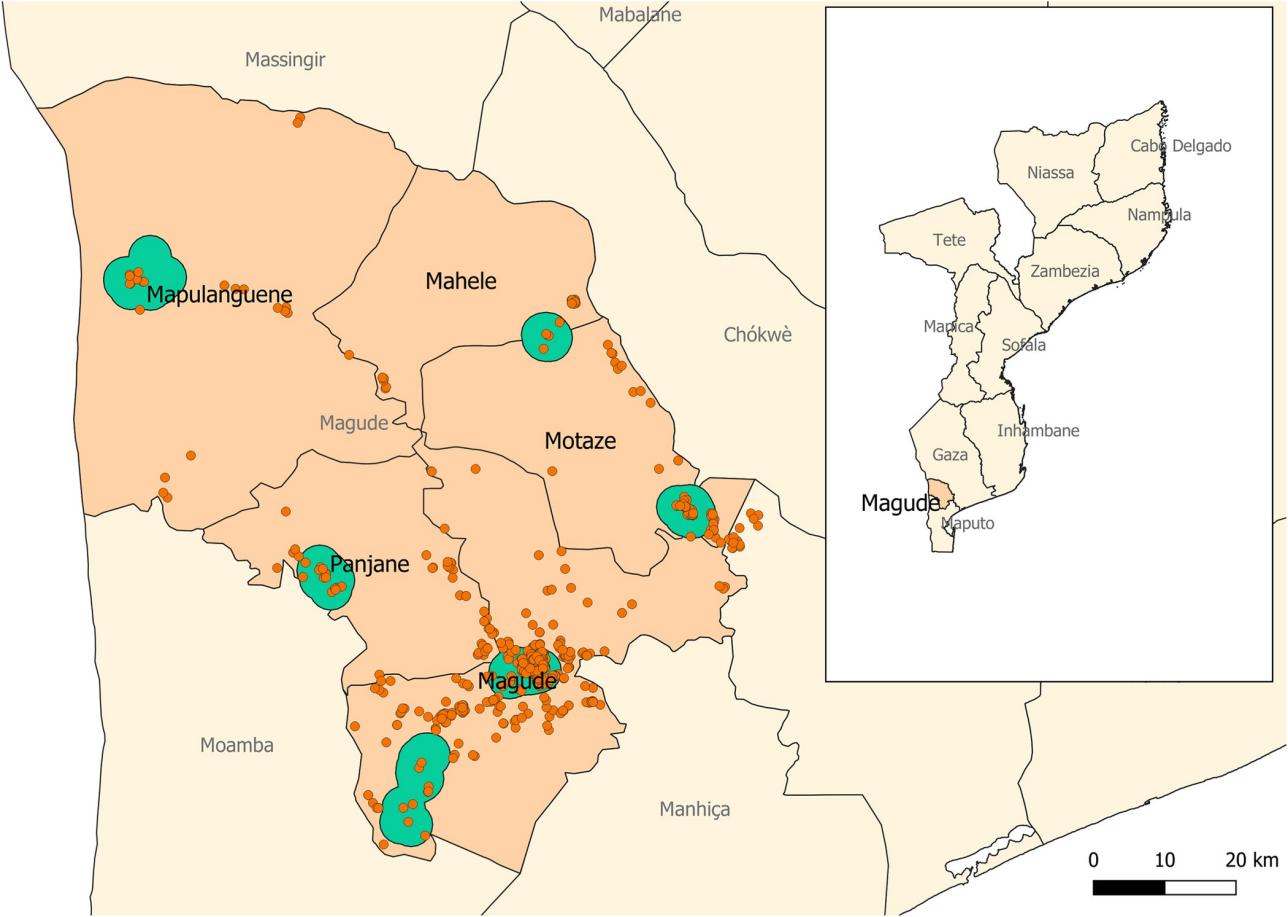
Map of Magude district. Red dots represent the households that were enrolled in the human behavioral study; the green areas are areas where entomological surveillance was conducted. The subnational administrative boundaries have been taken from the Humanitarian Data Exchange (https://data.humdata.org/dataset/cod-ab-moz) under a CC-BY-IGO license (https://data.humdata.org/faqs/licenses).

The study took place in Magude (Fig 1), a rural district located in Maputo Province, southern Mozambique. There were a recorded 48,448 residents in the district in 2015, and malaria prevalence by rapid diagnostic test ranged from 9.1% in May 2015 to 1.4% in May 2018. A comprehensive description of the district demographic, socio-economic and health characteristics is provided elsewhere [32].

The National Malaria Control Program distributed 35,432 LLINs during their mass distribution campaign in May 2014 [32]. In 2015, the percentage of households with at least one ITN for every two people was 53.2% [32]. The district received two rounds of IRS before and during this study, the first round (before our study) between August 2015 and October 2015 using dichlorodiphenyltrichloroethane (DDT) and pirimiphos-methyl (Actellic 300CS, Syngenta Crop Protection AG, Basel, Switzerland) and another round (during this study) between September 2016 and November 2016 using Actellic only. Apart from vector control activities, four rounds of mass drug administration (MDA) were implemented between 2015 and 2017. Based on data collected during the MDA campaigns immediately after the IRS rounds, 83% and 89.7% of the households were sprayed during 2015 and 2016, respectively [16,17]. More details on all interventions and their impact on malaria prevalence in the district are described elsewhere [17].

### Definition of transmission seasons

Based on a previous analysis showing that the incidence of malaria peaks two months after the peak rainfall [32], we considered the high transmission season to start in November (one month after the rainy season starts but one month before malaria peaks to account for immature mosquito development times) and the low transmission season in May (one month after the end of the rainy season, to account for mosquito longevity).

### Human behavior cross sectional evaluation

Human behavior was evaluated during both a low (17^th^ August to 2^nd^ November 2016) and a high transmission season (22^nd^ February to 19^th^ April 2017), based on the assumption that human behavior may differ between seasons due to e.g. climate conditions, perceived malaria risk and socio-economic activities. An age-stratified random sample of participants of three age groups (5 to 11 years, 12 to 17 years, and 18 years or older) was drawn from the district population using the population census database and respecting the proportion of people from each age group in each administrative division. The sample size allowed to estimate the percentage of exposure to host-seeking mosquitoes prevented by LLINs in each of the three age groups at 95% confidence with a 10% margin of error and it was calculated assuming a point estimate of 50% due to the lack of previous similar measurements in the country.

Human behavior was evaluated by means of close-ended structured interviews conducted by a trained field worker (S1 File). In addition, participants were given a digital watch (DigiTime DT23, Xonix Field Ranger or Xonix-BW007) and asked to record the actual time they (i) entered the house in the evening/night (time after which the participant did not go out of the house anymore), (ii) went to bed in the evening/night, (iii) got up in the morning and (iv) left the house in the morning, using a time-tracking card (S1 Fig). In both seasons, participants were visited during three consecutive days. On the first day, the field worker explained the study to the participants, obtained written informed consent from them or from their caretakers (for those in the 5–11 year-old group), provided the participants with a time-tracking card and a digital watch and instructed the participants how to complete the card using the watch. On the second and third day after this initial visit, the field worker conducted the structured interview and digitized the information from the participant’s time-tracking card. The first interview was considered a test round meant to ensure that participants had understood the use of the watch, the time-tracking card and the interview questions. During the structured interviews, participants were asked if they (i) used an LLIN to sleep the night before, (ii) used any other measures to prevent mosquito bites, (iii) left their bed during the night and (iv) worked at night. For the youngest age group (5–11 years old), their adult caretaker was asked to fill out the time-tracking card and respond to the survey questions on behalf of the child.

### Entomological surveillance

Vector surveillance started in May 2015, and data up to August 2017 have been included in the analysis to match the duration of the first phase of the Magude project. Mosquitoes were collected monthly in six sentinel sites in Magude district (Fig 1). In each sentinel site, mosquitoes were collected in fifteen representative houses during two consecutive nights every month. Collections took place indoors in 10 households and outdoors within the compound of 5 other households using miniature CDC light-trap (Model 512, John W Hock, Florida, USA). These traps were combined with Collection Bottle Rotators (Model 1512, John W Hock, Florida, USA) in 6 households (3 indoors; 3 outdoors, every night) to collect mosquitoes from time of trap placement to 6pm, and subsequently at 2h intervals until 6am, after which mosquitoes were collected in the final bottle until the team visited the house again. Every month, houses were randomly assigned a trap type (i.e. CDC-light trap with or without a rotator) and a collection environment (indoors or outdoors). Indoors, the CDC light-trap was placed at the foot-end of a bed with the trap opening approx. 1.5m above the ground. One or two adult volunteers (>15 years old) from the selected household were asked to sleep in the bed under an LLIN. Participants not owning a net were provided with a WHO-approved LLIN. Outdoors, CDC light traps were baited with a BG-Lure cartridge (Biogents AG, Germany) and CO_2_ that was generated through a mixture of 10g commercially available yeast (Instant Yeast, Smart Chef, Best Brands S.A., Tunesia), 100g white refined household sugar and 1L of regular tap water to mimic indoor conditions (i.e. a human sleeping next to the trap). The outdoor traps were placed in close proximity to the house with the trap opening approx. 1.5m above the ground, and were protected from the weather, theft, animals and/or children by available objects in the environment (mostly trees, or tall vegetation). Due to suspicion of arboviral diseases transmission in Mozambique, which has since been confirmed [33,34], no comparison against Human Landing Catches (HLC), the current gold standard methodology to assess human biting rates, were performed. As such, ‘exposure to hosts-seeking mosquitoes’ is reported throughout this study, rather than ‘vector bites’. Every morning after a collection night, the team visited the house to collect the mosquitoes and used a digital structured questionnaire to gather information on the collection conditions for data quality purposes (see data analysis section below).

Anopheline mosquitoes were identified morphologically to species using a stereomicroscope and the keys of Gillies and Coetzee [35]. Individuals belonging to the *Anopheles gambiae* s.l and *An*. *funestus* s.l complex were identified to species by multiplex polymerase chain reaction using the wing and leg [36,37]. *Plasmodium falciparum* sporozoites in mosquitoes were detected by means of enzyme-linked immunosorbent assay using the head and thorax of the mosquitoes [38].

### Data collection and analysis

Data from both studies were collected with tablets (Huawei, Model S7-701u) using Open Data Kit (https://opendatakit.org/). The analysis focused on evaluating the residual exposure of Magude residents to malaria vector species that survived the combined deployment of IRS and LLIN, the personal protection that LLINs conferred against exposure to host-seeking mosquitoes that survived or did not come in contact with IRS and LLIN products, and the personal protection they would have provided if all residents would have used a net. The exposure to residual host-seeking mosquitoes was quantified for five different environments where humans and mosquito vectors typically interact during the evening, night and early morning: i) outdoors, before people go indoors, ii) indoors, before people go to bed, iii) indoors, while people are in bed, iv) indoors, after people have gotten up, and v) outdoors, after people got up and left the house. Estimates are given for the two distinct malaria seasons: the low and high transmission season. We first analyzed the progression of our study participants through those environments, and the differences between seasons, age groups and gender. We then analyzed the host-seeking behavior of the local vector species during the low and high transmission seasons and subsequently overlapped both human and vector behaviors to obtain estimates of human exposure to host-seeking vectors in each environment and in both seasons. Finally, we estimated the proportion of exposure to the different host-seeking vector species that LLINs prevented through personal protection, and the proportion of exposure LLINs could have prevented if all residents would have used a net, again through personal protection. We compared LLIN personal protection across seasons and age groups.

### Human behavior

Only participants that reported sleeping indoors the night before the interview (99% of all participants) and who provided complete and chronologically consistent information for the time goings indoors, to bed, time of getting up and leaving the house were included in the analysis. The median time of the day at which participants went indoors, to bed, got up and left the house and the median amount of time they spent indoors (before going to bed, in bed, and after getting up) is reported together with the 90^th^ and 10^th^ quantiles to provide a measure of dispersion, since values were not normally distributed. Differences across seasons, age groups and gender were evaluated using the non-parametric tests (Mann-Whitney-Wilcoxon, Kruskal-Wallis rank sum test or Dunn’s Test for pairwise multiple comparison). The percentage of people who used an LLIN the night before the interview, used other mosquito protection measures and/or left the bed during the night was estimated and their 95% CI calculated using the normal approximation method. These percentages were compared across seasons, age groups and gender using Chi-square tests.

### Vector species composition, densities, sporozoite rates, and time of biting

Vector collections that met the exclusion criteria (S1 Box) were disregarded in the analysis. Species composition was estimated based on results of molecular species identification. Sporozoites in mosquitoes were detected by means of enzyme-linked immunosorbent assay [38]. Species composition and the number of host-seeking mosquitoes per person per time interval were calculated for the high and low transmission seasons separately. The number of host-seeking mosquitoes per person was calculated for each collection time interval by dividing the number of host-seeking mosquitoes collected at each time interval by the number of people sleeping in the room with the trap (or by one for outdoor collections) and by the minutes within the time interval. The rates obtained for each species and for each time period (e.g. 18:00–20:00) were averaged over a season to obtain season-representative values. The peak biting time of each species was considered to be the time interval with the highest rate of host-seeking mosquitoes per person.

### Exposure to host-seeking vectors adjusted for human behavior

The indicators used in the present analysis are an expansion of those proposed by Monroe *et al*. [24] and Killeen *et al*. [23]. All equations are provided in S1 Table. For each participant, we estimated the number of host-seeking mosquitoes that each participant is exposed to in each one minute intervals (B_*I*,*t*_, where *t* is expressed in minutes) through a modification of the method proposed by Killeen *et al*. [23]. We added the host-seeking mosquitoes per minute along the period of time that each participant spent in each environment to obtain the total residual exposure to host-seeking mosquitoes in each environment: outdoors before going indoors (B_*O*,*bb*_), indoors before going to bed (B_*I*,*bb*_), indoors and in bed (B_*I*,*b*_), indoors after getting up (B_*I*,*ab*_) and outdoors after getting up (B_*O*,*ab*_). For the purpose of calculating outdoor residual exposure to host-seeking mosquitos, we assumed that participants where outdoors between (i) 4pm (when mosquito collections started) and the time they reported going indoors, and (ii) between the time they reported leaving the house and 8am (when mosquito collections stopped). We assumed that participants were still exposed to host-seeking vectors while sleeping under a net, and that net users had an 81.1% reduction in exposure compared to people not using a net. This value is based on the percentage of mosquitoes that were observed to blood feed (18.9%) when participants in an experimental hut trial in Tanzania were sleeping under used Olyset® Nets (Sumitomo Chemical Company Ltd, Japan) [39]. We choose this value since i) Olyset Nets accounted for 77.1% of all nets in Magude district, ii) no local measurement on feeding inhibition were available, iii) it represented feeding inhibition of a mixture of wild *An*. *gambiae* and *An*. *funestus* mosquitoes (similar to our vector composition), and iv) the Olyset Nets in the trial had been in domestic use for 4 years and the Olyset Nets in Magude district were distributed approx. 2.3 to 3 years prior to this study. Observed feeding inhabitations with new but deliberately holed Olyset Nets were similar, with reported values of 82%, 83.8% and 84.2%, with the exception of a single study that reported 96.3% [39–42]. The limitations of the feeding inhibition parameter value are further explored in the discussion.

We estimated the proportion of residual exposure to host-seeking vectors in each environment both at the observed levels of bed net use and in the hypothetical situation that all residents used a net. To estimate the proportions, we summed the residual exposure to host-seeking mosquitoes experienced by all participants in a given environment and divided this value by the total across all environments. Proportions are reported with their 95% confidence intervals.

The proportions of residual exposure to host-seeking vectors occurring in the low transmission season at observed levels of bednet use (*π*_*r*,*low*_) and assuming all residents used a net to sleep (*π*_*p*,*low*_) were calculated by dividing the number of host-seeking vectors that all study participants were exposed to during the study night in the low transmission season by the number of host-seeking vectors they were exposed to during the low and high transmission seasons combined. This proportion was reported together with the 95% confidence interval using the normal approximation method.

### Actual and maximum personal protective efficacy of LLIN against host seeking vectors

The actual personal protection conferred by LLINs in Magude district ($P_{S,C}^{*}$) was calculated as the percentage of exposure to host-seeking vectors (that survived or did not come in contact with IRS and LLIN products) that LLINs prevented at the observed levels of bednet use: $P_{S,C}^{*}=100x(1-\frac{B_{r}}{B_{ru}})$, where B_*r*_ is the total number of host-seeking vectors that study participants were exposed to during one night at the observed levels of bednet use and B_*ru*_ the total number of host-seeking vectors that they would have been exposed to if none of them would have used nets to sleep. The 95% confidence intervals of $P_{S,C}^{*}$ were calculated using the normal approximation method.

The maximum personal protection that an LLIN could confer to each participant ($P_{S}^{*}$), i.e. the maximum percentage of exposure to host-seeking mosquitoes preventable through personal protection of a net, was estimated for each individual participant, rather than for the entire study population as other studies have proposed [23,24], to provide a more accurate measure of variability in the estimate. For each participant, we calculated $P_{S}^{*}=1-\frac{B_{p}}{B_{u}}$, where *B*_*p*_ is the total number of host-seeking vectors that the participant would have been exposed to if they used the bednet to sleep, and *B*_*u*_ if they did not use a bednet to sleep. Because we observed that the distribution of the individual $P_{S}^{*}$ was not normal (see S2 Fig) we reported median values plus their 10th and 90^th^ percentile for different seasons, age groups and species. Note that LLINs can provide community protection, whereby even community members who do not sleep under a net gain some protection due to reduction in the number of infected mosquitoes that are killed by LLINs that are used by other members. This community-level effect is however ignored in our analyses.

### Ethical clearance

Ethical approval was obtained from the Manhiça Health Research Center’s Institutional Bioethics Committee for Health (CIBS-CISM/072/2015 for our human behavior study; CIBS-CISM/043/2015 for our entomological surveillance) and local administrative authorities (52/SDSMASS/024.1). Before commencing any of the two studies, field workers informed participants of the objectives, risks and benefits of the studies, and how their data are protected and used, as well as of their right to withdraw from the study any time. For the human behavioral study, a written informed consent was provided and read out loud to all study participants. Only those that signed were enrolled in the study. Parents or official guardians signed the informed consent and responded to the survey on behalf of their children aged 5 to 11 years. Children between the age of 12 to 17 years provided consent themselves. For the entomological surveillance study, verbal informed consent was obtained from an adult member of the household to place the mosquito traps indoors or outdoors.

## Results

### Study participants

During the low transmission season, a total of 576 individuals were visited and 350 individuals were recruited of which 331 completed both interviews (168 women and 163 men). During the high transmission season survey, 536 individuals were visited, of which 331 individuals were recruited and completed both interviews (184 women and 147 men). The number of participants that slept indoors the night before the interview and that provided chronological values on their time-tracking card was 283 during the low and 289 during the high transmission season. The main reasons for unsuccessful visits included participants not being present at the time of the survey (53.5% of unsuccessful visits during the low and 44.4% during the high transmission season) followed by migration to other places (31.7% during the dry and 42.5% during the high transmission season). Very few participants rejected participation (3.3% during the low and 1.9% during the high transmission season). Ninety-nine percent of study participants slept indoors the night before.

### Bednet use

The percentage of people that slept under a bednet the night before the interview differed significantly between seasons (p<0.0001). In the high transmission season, LLIN use was 66.7% (95% CI: 60.4–72.9) whereas in the low transmission season use was 39.1% (95% CI: 30.7–47.6). Within each season, there was no significant difference in LLIN use between age groups or gender (χ^2^, p>0.05).

### Use of other measures to prevent mosquito bites

Study participants used additional measures to prevent mosquito bites beyond using an LLIN or living in a sprayed house. During the evening 52 individuals (13.9%) reported using clothing (60.8%), smoke (33.3%), charcoal (3.9%) or combining clothing and smoke (2%). During the night, 12 individuals (2.85%) reported using charcoal (33%), clothing (25%), smoke (25%) and commercial domestic insecticides (16.6%). During the morning, 6 (4.7%) individuals reported using clothing (83.3%) and smoke (16.7%). No differences were observed across age groups, sexes or betnet use (χ^2^, p>0.05).

### Human movement between environments: time going indoors, to bed, getting up and leaving the house again, and time spent indoors in bed and indoors before and after going to bed

During the low transmission season, the time (note all times reported here are medians) at which participants went inside was 19:40 and they spent 0.8h (p10^th^ = 0.09h, p90^th^ = 2.7h) indoors before going to bed. They went to bed at 20:37, stayed in bed for 9.4h (p10^th^ = 7.5h, p90^th^ = 10.8h) and got up at 06:10, after which they spent 0.3h (p10^th^ = 0.05h, p90^th^ = 1.11h) indoors before leaving the house at 06:35. The total time spent indoors not in bed was 1.4h (p10^th^ = 0.3h, p90^th^ = 3.5h). During the high transmission season, the time at which participants went indoors was 19:55, and they spent 0.6h (p10^th^ = 0.06h, p90^th^ = 1.9h) indoors before going to bed. They went to bed at 20:42, stayed in bed for 9.3h (p10^th^ = 7.4h, p90^th^ = 10.7h) and got up at 06:03, and spent another 0.3h (p10^th^ = 0.03h, p90^th^ = 1.16h) indoors before leaving the house at 06:30. The total time spent indoors not in bed was 1.1h (p10^th^ = 0.2h, p90^th^ = 2.9h). No significant differences were observed in these times between sexes. Values for different age groups and seasons with their statistical significant differences are shown in Table 1. Overall, in the low transmissions season, people went indoors earlier, spent more time indoors before going to bed and went to bed earlier than in the high transmission seasons (Mann–Whitney U, p<0.009). No significant differences were observed in the time spent in bed, the time at which participants got up, the time spent indoors after getting up and the time participants left the house.

**Table 1:** Median time of the day when participants went indoors, went to bed, got up and left the house after getting up, and the median amount of time they spent indoors before going to bed, in bed, and indoors after getting up before leaving the house. The letters (a,b,c) mark the pairs between which statistically significant differences were observed in pair-wise comparisons with Dunn Test. The * denotes that significant differences were found in all pair-wise comparisons with Dunn test (age groups) or Wilcoxon Mann Whitney (LLIN use).

	Time going indoors (HH:MM)		Time indoors before going to bed (h)		Time to bed (HH:MM)		Time in bed (h)		Time getting up (HH:MM)		Time indoors after getting up (h)		Time leaving house (HH:MM)		Total time indoors (h)		Total time indoors not in bed (h)	
	low	high	low	high	low	high	low	high	low	high	low	high	low	high	low	high	low	high
Age group																		
18+ years	19:30^a^	20:02^a^	1.3 ^ab^	0.9 ^ab^	21:00^b^	20:56 ^b^	8.7*	8.9 ^a^	05:57^ab^	05:49 ^ab^	0.4^a^	0.4	06:23^a^	06:16^a^	11^a^	10.2^a^	1.8*	1.4^ab^
12–17 years	19:59^ab^	20:01^b^	0.5 ^a^	0.5 ^a^	20:40^a^	20:47 ^a^	9.3*	9.2 ^b^	06:15^a^	06:04 ^a^	0.2^ab^	0.3	06:38	06:24	10.5^b^	10.4 ^b^	0.9*	1.1^a^
5–11 years	19:30^b^	19:34^ab^	0.8 ^b^	0.6 ^b^	20:17 ^ab^	20:20 ^ab^	9.9*	10 ^ab^	06:20^b^	06:10 ^b^	0.5^b^	0.3	06:46^a^	06:42^a^	11.5 ^ab^	11 ^ab^	1.4*	1^b^

Low: Low transmission season.

High: High transmissions season.

**Fig 2:**
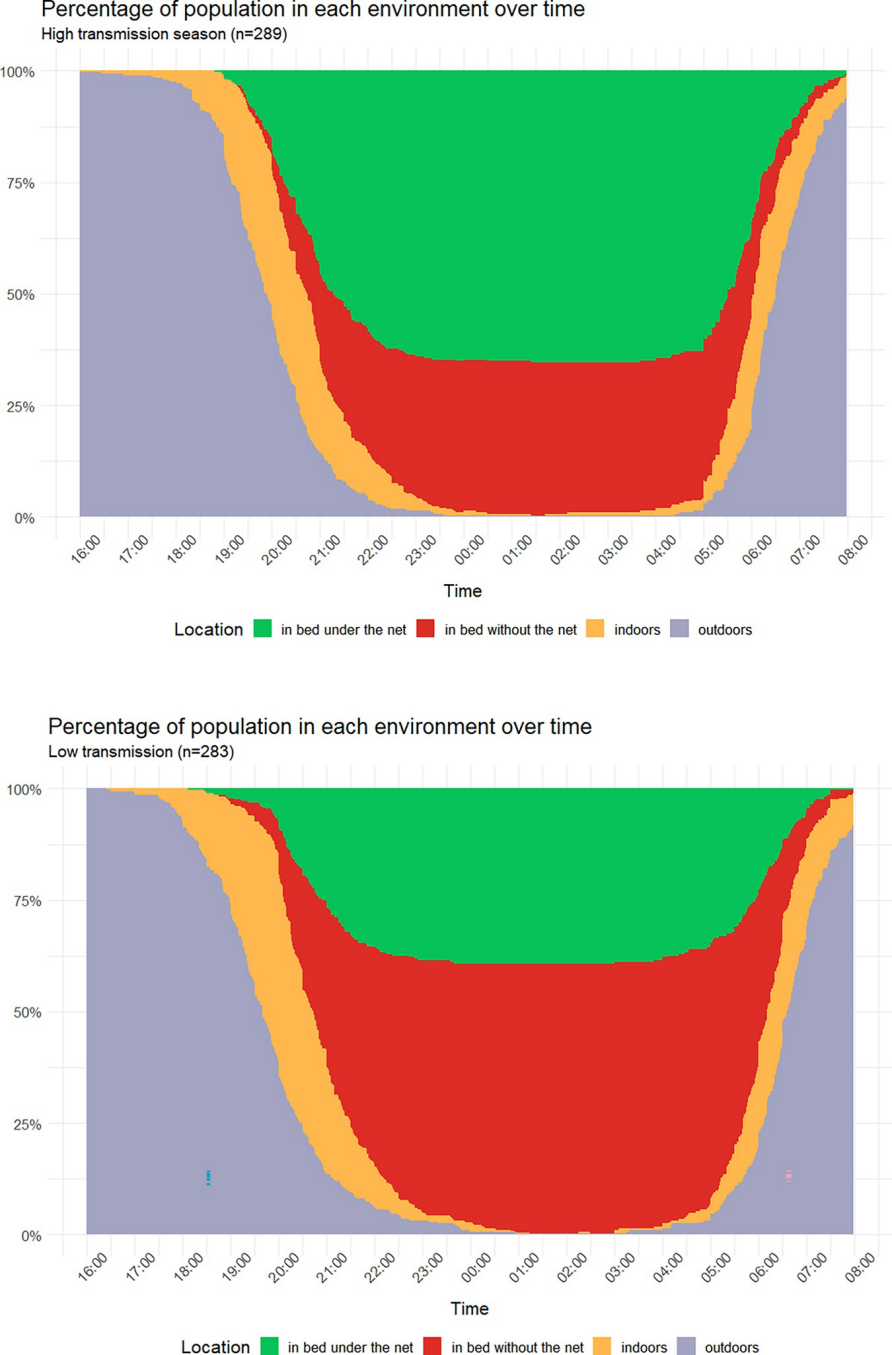
Percentage of study participants in each environment during the evening, night and morning. The environments show are: (i) outdoors before going indoors (grey area on the left-hand side), (ii) indoors but not in bed (yellow on the left-hand side), (iii) indoors in bed using an LLIN (green) or not using an LLIN (red), (iv) indoors but not in bed after getting up (yellow on the right-hand side), (iv) outdoors after getting up (grey area on the right-hand side), during the low transmission (left panel) and high transmission season (right panel). Data including the environment of the study participants after 8am can be found in S3 Fig.

To more easily pair the human behavioral data with the mosquito behavioral data (described below) that were collected in 2h intervals, we report the percentage of the study participants in the various environments during the same 2h time periods. The percentage of participants that were indoors by 18:00 was 2.4% in the high and 8.8% in the low transmission season. At 20:00 these values were 55.7% and 64.7%, respectively, and at 22:00 96.2% and 94%, respectively. The percentage of participants that was in bed by 20:00 was 24.2% in the high and 21.2% in the low transmission season. At 22:00 these values were 85.8% and 84.8%, respectively, and by midnight 99% and 97.2%, respectively. The distribution of the study participants in each environment over time is show in Fig 2.

### Differences in behavior time profiles across age groups and sex

In both seasons, children between 5 and 11 years of age spent more time indoors before going to bed, went to bed earlier, slept longer, got up later and left the house later than adults. In the high transmission season, they also went indoors earlier than adults. No differences were observed in the time spent indoors after getting up between children between 5 and 11 years of age and adults. In both seasons, children between 5 and 11 went indoors and to bed earlier and slept longer than children between 12 and 17. In the low transmission season they also spent more time indoors after getting up than children between 12 and 17 (Dunn, p<0.04). In both seasons, children between 12 and 17 spent less time indoors before going to bed and got up later than adults. In the low transmission season, they also went later indoors, slept less time and spent less time indoors after waking than adults.

### Participants leaving the bed during the night

The percentage of participants that left the bed during the night was significantly higher in the low transmissions season (32%; 106 participants) than in high transmissions season (21.1%; 70 participants) (χ^2^ = 58.407, df = 1, p <0.0001). The main reason was to go to the toilet (71.7% of adults, 94.6% of children between 12 and 17 years old and 96.6% of children between 5 and 11 years old), followed by taking care of babies (20.7% of the adults and 2.7% of the children between 12 and 17 years old). Toilets were mostly located outdoors (96.8%, 640 responses). This may result in additional exposures to indoor (childcare) and outdoor host-seeking mosquitos (toilet visit), but as this behavior was not assessed in greater detail, the exposure occurring during these times hasn’t been taken into account in the analyses below.

### Vector species composition, sporozoite rates and host-seeking times

A total of 4472 *Anopheles* female mosquitoes were collected between May 2015 and August 2017 in the CDC light trap collections (both in stand-alone traps and in those combined with the collection bottle rotator) and 3593 were analyzed for the presence of sporozoites. A total of 32 (0.9%) mosquitoes were sporozoite positive during the study period. Sporozoite rates per species during the study period were as follows: *An*. *squamosus* 5.8% (1/17), *An*. *funestus s*.*s*. 1.04% (1/96), *An*. *parensis* 1.0% (1/101) *and An*. *arabiensis* 0.9% (28/3021). Only *Anopheles* species found positive for *P*. *falciparum* malaria (i.e. incriminated as local vectors) were considered in the present analysis (we also included *An*. *merus*, as a positive specimen was found in September 2017). The majority of host-seeking anophelines of these five species collected (n = 3848) were *An*. *arabiensis* (81%; n = 3131) followed by *An*. *squamosus* (10%; n = 375), *An*. parensis (3%; n = 104), *An*. *merus* (3%; n = 130) and *An*. *funestus s*.*s*. (3%; n = 108). All *An*. *parensis* (except one individual) and more than two thirds of the *An*. *funestus s*.*s*. were collected during the low transmission season. No *An*. *parensis* were collected outdoors. *An*. *arabiensis*, *An*. *merus* and *An*. *squamosus* were more abundant during the high transmission season, when 70%, 61% and 88% of the mosquitoes were collected, respectively. No *Anopheles merus* were collected outdoors in any of the two seasons.

**Fig 3:**
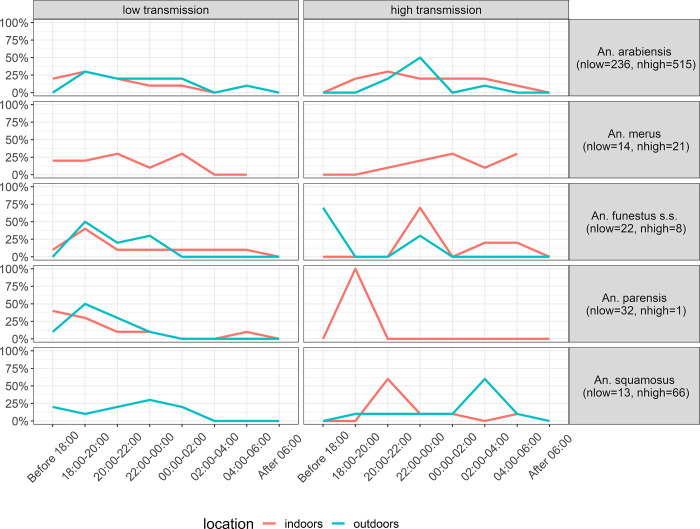
Host-seeking behavior of five different malaria vector species in Magude district between 4pm and 8am. The proportion of host-seeking mosquitoes collected indoors and outdoors is shown in 2 hour intervals.

The distinct host-seeking behavior of these five vectors is shown separately for the low and high transmission seasons in Fig 3. Overall, the peak of host-seeking activity occurred earlier in the low transmission season (between 18:00 and 20:00 indoors and outdoors) than in the high transmission season (between 20:00 and 22:00 indoors and 02:00–04:00 outdoors).

During the low transmission season, 8.7% of all outdoor host-seeking mosquitoes were collected before 18:00 (when most of participants were still outdoors, see above), and 50.7% before 20:00 (when half of the participants where still outdoors). Indoors, 20% of the host-seeking mosquitos were collected between 20:00 and 22:00 (whereby approx. half of the participants where already indoors and the other half moved indoors during this period) and 18.3% between 22:00 and 06:00 (when most of participants where in bed). During the high transmission, 4.9% of all outdoor host-seeking mosquitoes were collected before 18:00 (when most of participants were still outdoors), and 17.8% occurred before 20:00 (when half of the participants where still outdoors). Indoors, 9.5% of the host-seeking mosquitoes were collected between 20:00 and 22:00 (again with approx. half of the participants already being indoors and the other half moving indoors during this period) and 77.6% occurred between 22:00 and 06:00 (when most participants where in bed).

### Residual proportional exposure to host-seeking mosquitoes in each environment at the observed levels of bednet use in Magude district

Combining human and vector behaviors at the observed levels of bed net use and looking at both seasons combined, 74.0% (95% CI: 65-6-80.9) of all host-seeking mosquitoes that Magude residents were exposed to were *An*. *arabiensis*, 9.9% (95% CI: 4.8–13.2) *An*. *squamosus*, 5.8% (95% CI: 2.7–11.5) *An*. *parensis*, 5.2% (95% CI: 2.3–10.8) *An*. *merus* and 5.1% (95% CI: 2.2–10.6) *An*. *funestus s*.*s*.. Differences between seasons are shown in Table 2. Exposure to host-seeking *An*. *funestus s*.*s*. and *An*. *parensis* was higher during the low transmission season than in the high transmission season.

**Table 2:** The contribution of different vector species to the exposure to host-seeking vectors that Magude residents experienced. ‘Residual human-adjusted exposure to host-seeking vectors’ shows the percentage of host-seeking mosquitoes of each vector species that residents were exposed to at the observed levels of bednet use. ‘Unavertable residual human-adjusted exposure to host-seeking vectors’ shows the percentage of host-seeking mosquitoes from each vector species that residents would have been exposed to if they all would have used a net when in bed.

	Seasons combined		Low transmission season		High transmission season	
Species	Actual human-adjusted exposure to host-seeking vectors (%)	Unavertable human-adjusted exposure to host-seeking vectors (%)	Actual human-adjusted exposure to host-seeking vectors (%)	Unavertable human-adjusted exposure to host-seeking vectors (%)	Actual human-adjusted exposure to host-seeking vectors (%)	Unavertable human-adjusted exposure to host-seeking vectors (%)
***An*. *arabiensis*** *(n = 751*, *nlow = 236*, *nhigh = 515)*	74.0% (65-6-80.9)	69.2% (65.6–80.9)	64.2% (48.3–77.6)	56.6% (36.8–74.6)	78.5% (68.6–86.1	75.5% (61.8–85.7)
***An*. *squamosus*** *(n = 79*, *nlow = 13*, *nhigh = 66)*	9.9% (4.8–13.2),	12.8% (6.7–22.3)	5.1% (10.2–17.4)	8.2% (16.5–26.5)	12.2% (6.6–21.0)	15% (7.2–27.8)
***An*. *parensis*** *(n = 33*,*nlow = 32*, *nhigh = 1)*	5.8% (2.7–11.5)	7.4% (3.1–15.9)	17.8% (8.5–32.7)	22.0% (9.3–42.2)	0.1% (0–5.0)	0.1% (0–8.3)
***An*. *merus*** *(n = 35*, *nlow = 14*, *nhigh = 21)*	5.2% (2.3–10.8)	4.2% (1.2–11.8)	4.1% (0.6–16.0)	3.1% (0.9–19.7)	5.8% (2.2–13.2)	4.8% (1.1–15.2)
***An*. *funestus s*.*s*.** *(n = 30*, *nlow = 22*, *nhigh = 8)*	5.1% (2.2-10-6).	6.4% (2.4–14.6)	8.8% (2.8–22.)	10.1% (2.5–28.8)	3.3% (0.8–9.9)	4.5% (0.09–14.7)
**All species (n = 928, nlow = 317, nhigh = 611)**	100%	100%	100%	100%	100%	100%

Looking at the risk per environment, combining both seasons, the majority of residual exposure to host-seeking mosquitoes was estimated to occur when people where in bed (64%, 95% CI: 55.3–1.9), followed by indoors before going to bed (21.9%, 95% CI: 15.5–29.9), outdoors in the evening (12.5%, 95% CI: 7.7–19.5), indoors after getting up (1.4%, 95% CI: 0.2–5.6) and outdoors during the morning (0.2%, 95% CI: 0–3.8). Of the residual exposure, 32.4% (95% CI: 24.7–40.9) occurred during the low transmission season and 66.7% (95% CI: 59.0–75.2) during the high transmission season. A higher proportion of residual exposure occurred outdoors and indoors while not in bed in the low transmission season, compared to the high transmission season (Table 3).

**Table 3:** Percentage of host seeking mosquitoes of each of the five vector species that Magude residents were exposed to in each of the five environments where humans and vectors have the opportunity to interact. These environments are i) outdoors before going indoors, ii) indoors before going to bed, iii) indoors while in bed, iv) indoors after getting up, and v) outdoors after leaving the house again. Percentages are given for the low and high transmission seasons separately, and for the observed levels of bednet use, or assuming a hypothetical scenario in which all residents used a net when in bed.

	Low transmission season						High transmission season				
	Outdoors evening	Indoors before going to bed	Indoors while in bed	Indoors after getting up	Outdoors morning		Outdoors evening	Indoors before going to bed	Indoors while in bed	Indoors after getting up	Outdoors morning
**% of host-seeking mosquitoes at observed levels of bednet use**											
*An*. *arabiensis* *(n = 236)*	5.6% (0.7–22.8)	30.3% (15.3–50.4)	63%% (43–79.6)	1.0% (0,16.3)	0.1% (0–15)	*An*. *arabiensis* *(n = 515)*	3.4% (0.8–11.4)	21.6% (13.2–33.1)	73.4% (61.5–82.7)	1.5% (0.1–8.6)	0.1% (0–6.4)
*An*. *merus* *(n = 14)*	0.0% (0–82.5)	25% (0–91.2)	75.0% (8.8–100)	0.0% (0–82.5)	0.% (0–82.5)	*An*. *merus* *(n = 21)*	0.0% (0–51.6)	9.7% (0–60.4)	85.3% (35.4–99.6)	5.1% (0–56.4)	0.0 (0–51.6)
*An*. *funestus s*.*s* *(n = 22)*	40.1% (6.2–85.2)	19.8% (0.4–75.5)	39.2% (5.6–85.2)	0.9% (0–62)	0.0% (0–61.3)	*An*. *funestus s*.*s* *(n = 8)*	63.4% (12.7–96.7)	2.2% (0–69.8)	33.3% (1.9–87.1)	1.2% (0–69.1)	0.0% (0–68.3)
*An*. *parensis* *(n = 32)*	49.5% (20.7–78.6)	17.7% (1.9,58.5)	32.1% (7.5–70.3)	0.6% (0–41.3)	0.0% (0–40.6)	*An*. *parensis* *(n = 1)*	0.0% (0–99.5)	93.1 (0.5–100)	6.9% (0–99.5)	0.0% (0–99.5%)	0.0 (0–99.5)
*An*. *squamosus* *(n = 13)*	100% (23.1–100)	0.0% (0–76.9)	0.0% (0–76.9)	0.0% (0–76.9)	0.0% (0–76.9)	*An*. *squamosus* *(n = 66)*	29.4% (8.6–62.1)	20.9% (4.4–54.3)	46.3% (19.6–75.2)	1.2% (0–33.1)	2.2 (0–34.3)
Species combined	21.1% (10.9–36.4	25.4% (12–21)	52.7% (37.3–67.6)	0.8% (0–11.2)	0.1% (0–10.1)	Species combined	8.4% (3.9–16.5)	20.3% (12.9–30.1)	69.4% (58.8–78.3	1.6% (0.2–7.6)	0.3% (0–5.5)
**% of host-seeking mosquitoes assuming all residents used the net**											
*An*. *arabiensis* *(n = 236)*	10.3% (1.3–37.4)	55.1% (29.4–78.6)	32.6% (12.8,60.1)	1.8% (0–27)	0.3% (0–24.9)	*An*. *arabiensis* *(n = 515)*	6.0% (1.3,19.2)	37.9% (23.8–54.3)	53.3% (37.4,68.6	2.6% (0.2,14.5)	0.1% (0–19.7)
*An*. *merus* *(nl = 14)*	0% (0–95.3)	54.1% (4.7–96.6)	49.5% (3.4–95.3)	0.0% (0–95.3)	0.0% (0–95.3)	*An*. *merus* *(n = 21)*	0% (0–72.5)	19.5% (0–83.3)	70.2% (12.1–99.3)	10.2% (0–78.5)	0.0% (0–72.5)
*An*. *funestus s*.*s* *(n = 22)*	55.8% (12–92.5)	27.6% (0.6–86.1)	15.4% (0–80.1)	1.3% (0–71.9)	0.0% (0–71.1)	*An*. *funestus s*.*s* *(n = 8)*	78.9% (14.6–100)	2.7% (0–76.1)	17.0% (0–83.5)	1.4% (0–75.4)	0.0% (0–74.6)
*An*. *parensis* *(n = 32)*	64.3% (22.7–92.9)	23.0% (2.5–68.3)	11.9 (0.2–59.2)	0.7% (0–40.9)	0.8% (0–48.8)	*An*. *parensis* *(n = 1)*	0.0 (0–99.5)	95.7% (0.5–100)	4.3% (0–99.5)	0.0% (0–99.5)	0.0% (0–99.5)
*An*. *squamosus* *(n = 13)*	100% (23.1–100)	0.0% (0–76.9)	0.0% (0–76.9)	0.8% (0–48.8)	0.0% (0–76.9)	*An*. *squamosus* *(n = 66)*	40.2% (12–75.6)	28.5% (6.1–66.7)	26.0% (5.3–65.1)	1.7% (0–42.7)	3.0% (0–42.7)
Species combined	33.8 (17.7–54.2)	40.7% (23.2–60.7)	24.0% (10.7–44.4)	0.0% (0–76.9)	0.2% (0–15.4)	Species combined	14.1% (6.7–26.8)	34.1% (22.2–48.2)	48.4% (34.9-,62.1)	2.8% (0.3–12.4	0.5% (0–9)

### Proportion of exposure to host-seeking vectors prevented by the personal protection of LLINs in Magude district

**Fig 4:**
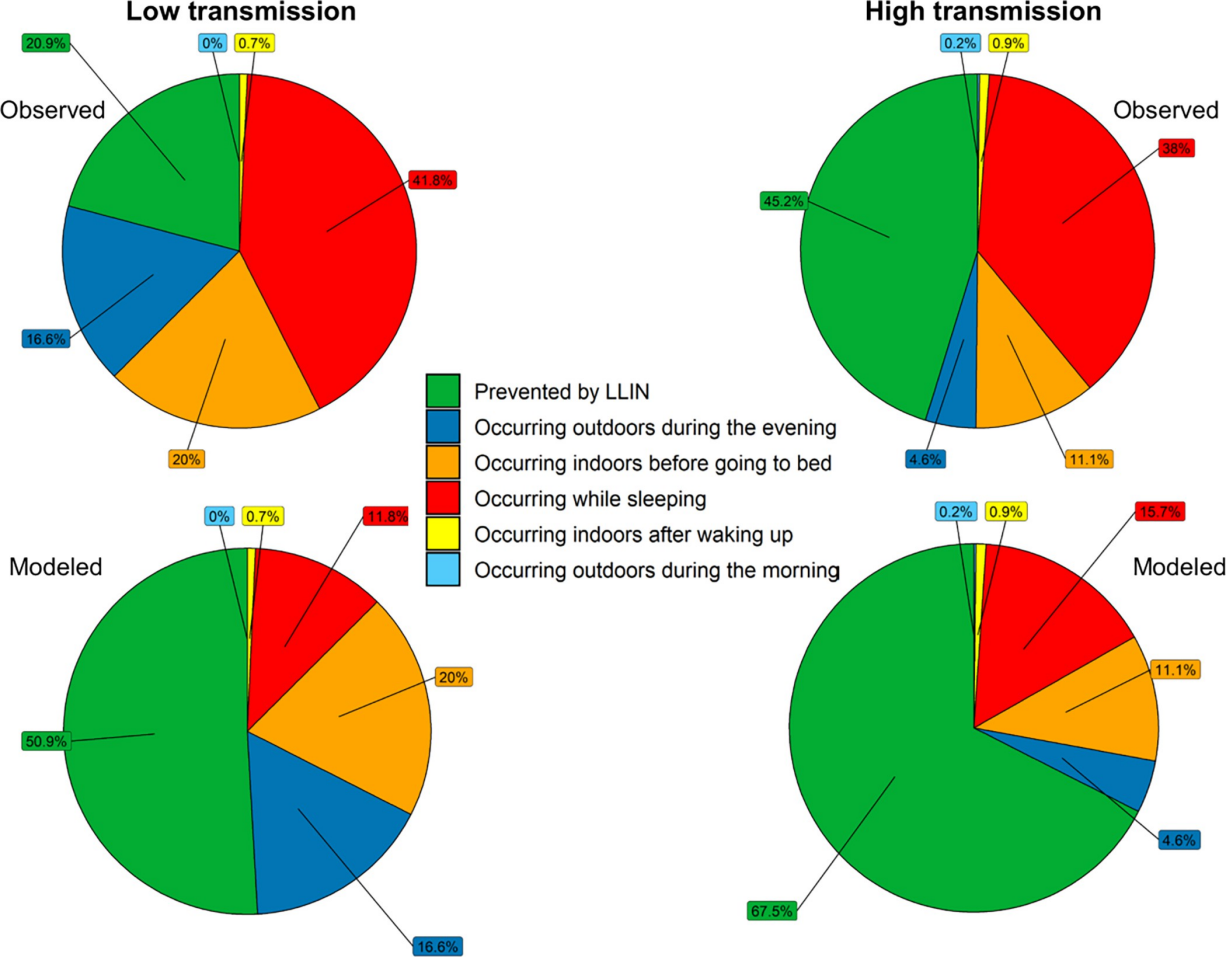
The proportion of exposure to host-seeking mosquitoes prevented by the personal protection of LLINs and the distribution of the unprevented exposure across the five different environments. The proportion of residual exposure to host-seeking vectors are provided at the observed (top) and modeled (bottom) net use (assuming all residents use a net while in bed). Green: exposure prevented by LLINs, dark blue: residual exposure outdoors before going indoors, orange: residual exposure indoors before going to bed, red: residual exposure while in bed, yellow: residual exposure indoors after getting up, light blue: residual exposure outdoors after leaving the house again.

At the observed levels of bednet use and considering both seasons together, the personal protection of LLINs averted 39.2% (95% CI: 32.8–45.9) of the exposure to host-seeking mosquitoes that survived or did not come in contact with IRS and LLIN products. This percentage was lower in the low transmission seasons (20.9%, 95% CI: 11.6–34.2) than in the high transmission season (45.3%; 95% CI: 37.7–53.1). A comparison between the proportion of exposure prevented by the personal protection of LLINs and that still occurring in the different environments is shown in Fig 4 for each season. LLINs prevented a significant higher proportion of exposure in children between the age of 5 and 11 (45.4%) than in children between the age of 11 and 17 (32.5%) or adults (38.9%). Statistically significant differences were also observed in the proportion of exposure prevented against different vector species (Kruskal-Wallis, p<0.0001). LLINs prevented a higher proportion of exposure to host-seeking members of the *An*. *gambiae* group (41.8% [95% CI: 34.4–49.5] for *An*. *arabiensis* and 45.4% [95% CI: 20,73.2] for *An*. *merus*) than from *An*. *squamosus* [32.0%, 95% CI: 14.2–56.3] and members of the *An*. *funestus* group (21.9% [95% CI: 3.8–59.7] for *An*. *funestus s*.*s*. and 13.9% [95% CI: 1.3–51.5] for *An*. *parensis*).

### Maximum personal protection that LLINs could have conferred in Magude district

Considering both seasons combined, the maximum proportion of exposure to host-seeking mosquitoes that the personal protection of LLINs could have averted if all residents would have used a net while in bed, assuming that an increase in net use would not have led to an immediate change in vector host-seeking behaviors (see discussion) was 63.3% (p10th = 41.2, p90th = 75.2; Fig 4). This was lower during the low transmission season (50.7%, p10th = 35.6, p90th = 62.6) than during the high transmission season (67.5%, p10th = 53.8, p90th = 76.5).

The potential personal protection that LLINs could have provided if all residents would have used a net considering both seasons was significantly different between age groups (Kruskal-Wallis, p<0.0001). This maximum personal protective efficacy would have been lowest for adults (57.0%; p10th = 35.6, p90th = 73.2) and highest for children between 5 and 11 years of age (62.5%; p10th = 47, p90th = 75.8), but would not differ between the youngest and oldest child groups (61.0%; p10th = 43.4, p90th = 75.7).

The maximum personal protection that LLINs could have conferred would also have differed significantly between the different vector species (Kruskal-Wallis, df = 4, p<0.0001). LLINs would have prevented a higher proportion of exposure to host-seeking vectors of the *An*. *gambiae* group than to those of the *An*. *funestus* group, similar to the results of the previous section. Regarding the individual species, LLINs would have prevented a higher percentage of exposure to host-seeking *An*. *arabiensis* (67.4%; p10th = 45.9, p90th = 77.4), followed by *An*. *merus* (74.8%, p10th = 55.3, p90th = 80.5), *An*. *squamosus* (19.8%; p10th = 0, p90th = 66.6), *An*. *funestus s*.*s*. (42.6%; p10th = 32.1, p90th = 44.5) and *An*. *parensis* (31.7%, p10th = 0, p90th = 47.3).

### Residual exposure to host-seeking vectors that would occur if all residents of Magude would have slept under the net every night

Of the residual exposure to host-seeking vectors that still would have occurred if all residents would have used a net while in bed, 40.3% (95% CI: 29.8–51.6) would have happened indoors while participants are in bed due to the imperfect feeding inhibition of LLINs (see discussion), followed by 36.3% (95% CI: 26.2–47.7) indoors before going to bed, 20.7% (95% CI: 12.9–31.3) outdoors during the evening, 2.3% (95% CI: 0.4–9.1) indoors after getting up and 0.4% (0–6.2) outdoors after getting up and leaving the house. Overall, 33.4% (95% CI: 23.6–44.7) of this exposure would have occurred during the low transmission season and 66.6% (55.2–76.3) during the high transmission season. In this scenario, the contribution of members of the *An*. *funestus* group and of *An*. *squamosus* would have been higher than at the observed levels of bednet use (Table 3).

## Discussion

We aimed to understand (i) the residual malaria transmission that occurred during the Magude project by characterizing residual exposure to host-seeking vectors occurring when LLINs and IRS were deployed together, (ii) the protection that LLINs conferred, and could have conferred if all residents would have used a net to sleep, against exposure to host-seeking vectors, and (iii) the residual exposure to host-seeking vectors that would have occurred even if all residents would have used a net. We hope our results help to optimize the implementation of current tools and guide the development and implementation of supplementary vector control interventions in low transmission settings in sub-Saharan Africa.

*An*. *arabiensis* was responsible for more than 74% of residual exposure to host-seeking vectors experienced by Magude residents during the Magude project. The role of *An*. *arabiensis* as the potential main driver of residual malaria transmission after the implementation of district-wide IRS campaigns has repeatedly been observed in southern and eastern African countries [43–45]. The other four vector species (*An*. *funestus s*.*s*., *An*. *parensis*, *An*. *squamosus* and *An*. *merus*) were each responsible for less than 10% of the residual exposure to host-seeking vectors. At the observed level of bednet used, 12.5% of residual exposure occurred outdoor during the evening, 21.9% indoor before going to bed, almost two thirds (64%) while people were in bed, 1.4% indoors after getting up and 0.2% outdoor after leaving the house. Almost a third of the exposure (32%) occurred during the low transmission season. The personal protection conferred by LLINs prevented only 39.2% of the exposure to the host-seeking vectors that survived or did not come in contact with IRS and LLIN products during the Magude project, and could have prevented a maximum of 63.3% if all residents would have used an LLIN to sleep (assuming that the increase in LLIN use does not lead to an immediate change in vector host-seeking behavior). The maximum personal protection nets could have provided differed across seasons, vector species and age groups. The personal protection of LLINs prevented a higher proportion of the exposure to host-seeking vectors of the *An*. *gambiae* group than to those of the *An*. *funestus* group, and provided better protection among children between 5 and 11 years compared to other age groups, and in the high compared to the low transmission season.

During phase I of the Magude project, residual exposure to host-seeking vectors from all vector species occurred mainly indoors (87.3%), primarily while people were in bed (64%). The latter is mainly due to the observed levels of bed net use as well as the estimated proportion of bites still occurring while people are under a net (due to the imperfect feeding inhibition of LLINs assumed in our calculations). If all Magude residents would have used a net to sleep (again assuming that the increase in LLIN use does not lead to an immediate change in vector host-seeking behavior), our estimates indicate that the personal protecting effect of LLINs alone would have prevented an additional 24.1% of exposure to host-seeking mosquitoes that survived or did not come in contact with IRS and LLIN products.

In the hypothetical scenario that everyone would have used a net, and again assuming that an increase in LLIN use does not lead to an immediate change in vector host-seeking behavior, the highest proportion of residual exposure to host-seeking mosquitoes would still occur indoors (78.9%), and also when people are in bed (40.3%). This suggests that large gains to further reduce transmission in settings where *An*. *arabiensis* is the predominant residual malaria vector could be achieved by increasing the feeding inhibition of LLINs and from additional vector control interventions that reduce the indoor human-vector contact.

In contrast, our analysis of the limited number of members of the *An*. *funestus* group suggests that the highest proportion of residual exposure to *An*. *funestus s*.*s*. and *An*. *parensis* occurred outdoors during the evening, which explains the low personal protection conferred by LLINs against exposure to these species. This observed behavior could be a result of selection pressure exerted by the continuous historical implementation of insecticide-based vector control interventions, which has been observed elsewhere to shift vector behaviors to outdoor feeding [46]. Although these results will need to be confirmed by additional studies, supplementary interventions that aim to reduce the densities of outdoor biting vector populations (e.g. through larval source management or attractive targeted sugar baits) or prevent outdoor human-vector interactions (e.g. through topical repellents or impregnated clothing) will be needed to reduce the residual exposure to *An*. *funestus s*.*l*. Note that for all of the five mosquito species that we analyzed, the latter interventions are mostly needed during the evening hours (before midnight), as the proportion of residual exposure to these vectors during the early morning was very small.

LLINs provided less personal protection during the low transmission season, when almost a third of the overall exposure to host-seeking vectors recorded in Magude district occurred. This was mainly driven by the lower LLIN use, but also by the earlier vector host-seeking activity observed during this season. The seasonal variation in bednet use has been observed in several other countries [47] and highlights the need to increase LLIN use during this particular season, as malaria transmission can still persist. Additional interventions are needed to tackle the problem of early host-seeking vectors during the dry transmission season, both outdoors and indoors before people go to bed. In addition, the fact that higher number of *An*. *funestus s*.*s*. and *An*. *parensis* were collected during this season compared to the high transmission season, suggests that these interventions could have a great impact in reducing the abundance of members of the *An*. *funestus* complex.

Since there were no differences in LLIN use between age groups, the difference in personal protection by LLINs observed between the age groups is due to differences in human behavior. The fact that young children went to bed earlier and slept longer in both seasons, and that they went indoors earlier during the high transmission season, means that the personal protection conferred to them by LLINs probably prevented a higher proportion of the exposure to host-seeking vectors in this age group than for the other age groups. This, and the fact that such behaviors can differ between regions (e.g. residents in Tengua, Milange district, Mozambique, went indoors and to bed later, slept less and got up earlier than people in Magude [48,49]) highlight the importance of collecting local human behavioral data to accurately estimate transmission risk and the protective efficacy of LLINs, but also of other tools that aim to reduce vector-host contact.

The low number of sporozoite positive mosquitoes and the lack of data on mosquito blood meal sources prevent us from estimating the Entomological Inoculation Rate and thus from drawing firm conclusions on the relative importance of each species in sustaining residual malaria transmission during the Magude project. Nonetheless, our results do suggest that at least five species were potentially contributing to sustaining transmission during the Magude project (*An*. *arabiensis*, *An*. *funestus s*.*s*., *An*. *parensis*, *An*. *squamosus* and *An*. *merus*) and that their contribution differs between the studied environments in which people and mosquitoes interact. Transmission by *An*. *funestus s*.*s*. and *An*. *parensis* is more likely to have occurred outdoors and indoors before people go to bed, while *An*. *arabiensis* and *An*. *merus* fed commonly indoors when people are in bed. Transmission by *An*. *squamosus* likely occurred both before people go to bed and while people are in bed. Although -based on the percentage of residual exposure attributed to each species- *An*. *arabiensis* may seem the most important vector of transmission, the fact that *An*. *funestus s*.*s*. can still drive transmission even if it is less abundant than *An*. *arabiensis* [50] suggests that *An*. *funestus* may still have played an important role in sustaining local malaria transmission during the Magude project.

There are, however, some limitations of the present study that may affect the accuracy of our estimations of exposure to host-seeking mosquitoes and of the personal protective efficacy of LLINs. First, due to the overall low baseline malaria prevalence and the four rounds of MDA conducted during the Magude project, very few mosquitoes were found positive for *P*. *falciparum*. As such, we may have excluded vectors species in our analysis. Secondly, our outdoor CDC Light traps were baited with a BG-lure (containing artificial skin compound mimics) and CO_2_ to simulate a human host, but we did not validate these CDC light traps collections with CDC light traps with an actual human bait present outside. Differences in sampling efficacy may lead to changes in the proportion of host-seeking mosquitoes collected outdoors and to the over- or underestimation of the importance of this transmission environment. Thirdly, our analyses are based on participants self-reported behaviors and timings and may therefore be affected by an incapacity to properly use the digital watch provided to them, have difficulties in reading or interpreting the time recording cards, have a response-bias (e.g. claiming using the net when they did not) or a recall bias, although the latter is expected to be minimal since participants were asked in the morning about their behaviors during the previous night. Fourthly, we showed that human behaviors differ between seasons but assumed that the respective behaviors remained similar during all low and all high transmissions seasons across different years during the Magude project. Yet, there may have been unaccounted changes in human exposure to host-seeking mosquitoes due to e.g. increased awareness of malaria and/or mosquitoes during the Magude project, or exceptionally dry and wet years. Or we may have missed short-termed intra-season events that may increase exposure to host-seeking mosquitoes (e.g. those linked to agricultural activities). Fifth, the exposure experienced by the one percent of participants that did not sleep indoors or the exposure during the night when the study participants had to get up for childcare and/or to go to the toilet (between 21% and 32% of the participants) were not considered, but those behaviors could increase the overall exposure to host seeking mosquitoes. In addition, the temporal resolution of the vector behavioral data (2h) compared to the human behavioral data (1min) limit the accuracy of the estimates. However, it was sufficient to detect significant differences in LLIN protection across age groups and seasons.

The final limitation deserves special attention and a call for action. Estimates of the protective efficacy of LLINs and residual exposure to host-seeking mosquitoes are sensitive to the LLIN blood feeding inhibition chosen. As stated in the methods, we assumed an 81.1% reduction in exposure to host seeking mosquitoes when participants were under a used Olyset® Net (the main net brand observed in the district). This value is based on a study conducted in Tanzania [39], because local measurements of net feeding inhibition were not available. Data on Olyset® net feeding inhibition are available from a limited number of countries, mostly located in West Africa (Benin, Burkina Faso, Cote D’Ivoire, Nigeria) and one in east Africa (Tanzania) [51]. It is common to use the data obtained in those few countries, or to use an arbitrary value, when estimating the personal protection of LLINs in one’s own country [26,28,52]. We selected 81.1% from all published values from Tanzania, because the experimental conditions represented the local conditions in Magude best (mosquito species composition, prior net use, see methods). This value was the lowest among all published values (except for those values for Olyset Nets used for 7 years), and therefore generates the most conservative estimates for the protective efficacy of LLINs during the Magude project. However, a wide range of feeding inhibition values has been observed across different experimental hut trials with the same net brand, and between different vector species [51]. Therefore local measurements of the LLIN feeding inhibition against local vector species are needed to i) accurately quantify the protective efficacy of nets, and ii) evaluate the residual exposure to vector bites after deployment of interventions, to better understand the gaps in the protection by LLINs.

## Conclusion

The combined deployment of IRS and LLINs during the Magude project was not sufficient to prevent all malaria vector bites. The residual exposure to *An*. *arabiensis* indoors when people are in bed, *An*. *funestus s*.*s*. and *An*. *parensis* outdoors and indoors before bedtime, and of *An*. *squamosus* both indoors and outdoors, are likely to have sustained malaria transmission throughout the Magude project. The low transmission season should not be neglected when implementing vector control interventions during malaria elimination campaigns, as this season accounted for a third of the residents’ total exposure to host-seeking mosquitoes. In areas where the main malaria vector feeds indoors while people are in bed, like *An*. *arabiensis* in this study, increasing bednet use and net feeding inhibition (e.g. by improving LLIN quality and/or selecting LLIN brands after a local evaluation), can lead to significant reductions in exposure to host-seeking vectors and likely further reduce malaria transmission. However, supplementary interventions aiming to reduce human-vector contact outdoors and/or indoors before people go to bed (e.g. through larval source management, window and eave screening, eave tubes, and spatial repellents) will be needed to reduce residual biting by outdoor and earlier biting vectors such as *An*. *funestus s*.*s*. and *An parensis*.

## Supporting information

S1 Fig Time-tracking card provided to each study participant to track movement between compartments. Participant were asked to record the following: (i) time going indoors, (ii) time going to bed, (iii) time getting up and (iv) time leaving the house in the morning.
(DOCX)

S2 FigDistribution of individually calculated $P_{S}^{*}$.This is the maximum percentage of exposure to host-seeking mosquitoes that LLINs could have prevented for each individual (through personal protection) if they would have used the net while in bed.(DOCX)

S3 Fig Location of study participants during the evening, night and morning. Percentage of participants that were outdoors (grey area), indoors but not in bed (yellow), indoors in bed using an LLIN (green) and indoors in bed but not using an LLIN (red) during the low transmission (left panel) and high transmission season (right panel), including human behavioral data recorded after 8am.
(DOCX)

S1 Table Model parameters with their definitions and equations. (DOCX)

S1 Box Exclusions criteria for mosquito surveillance data. (DOCX)

S1 File Structured questionnaire to assess human behaviors. (DOCX)

S2 File Human behavior dataset. (XLSX)

S3 File Mosquito surveillance dataset. (XLSX)
