# Supplementary material for: Overlaying human and mosquito behavioral data to estimate residual exposure to host-seeking mosquitoes and the protection of bednets in a malaria elimination setting where indoor residual spraying and nets were deployed together
Source: PLoS One. 2022 Sep 15;17(9):e0270882. doi: 10.1371/journal.pone.0270882 (PMC9477321; doi:10.1371/journal.pone.0270882)

**S1 Fig. Time-tracking card provided to each study participant to track movement between compartments.** Participant were asked to record the following: (i) time going indoors, (ii) time going to bed, (iii) time getting up and (iv) time leaving the house in the morning.

Original version (Portuguese)


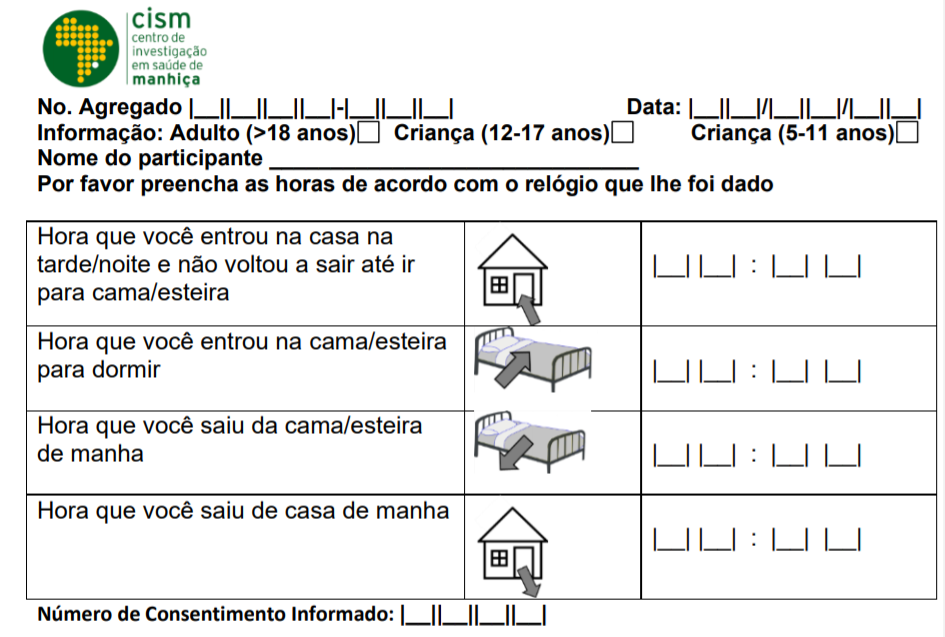


Translated version (English)


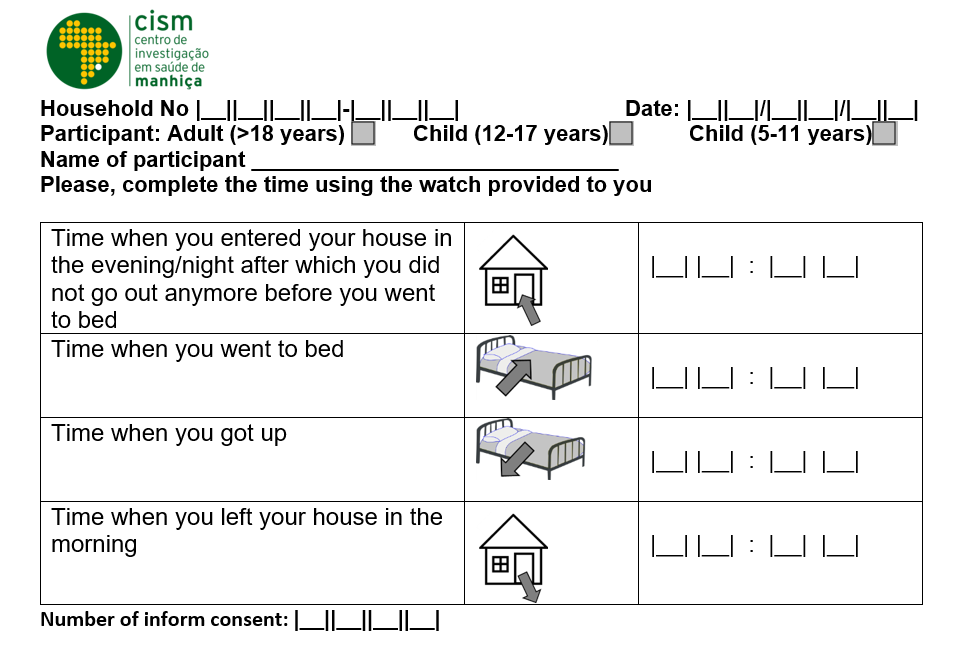

Supplement: S1 Fig — Participant were asked to record the following: (i) time going indoors, (ii) time going to bed, (iii) time getting up and (iv) time leaving the house in the morning. (DOCX) [file pone.0270882.s001.docx]
