# Supplementary material for: Overlaying human and mosquito behavioral data to estimate residual exposure to host-seeking mosquitoes and the protection of bednets in a malaria elimination setting where indoor residual spraying and nets were deployed together
Source: PLoS One. 2022 Sep 15;17(9):e0270882. doi: 10.1371/journal.pone.0270882 (PMC9477321; doi:10.1371/journal.pone.0270882)

**S4 Fig. Distribution of individually calculated** $\boldsymbol{P}_{\boldsymbol{S}}^{\boldsymbol{*}}$**.** This is the maximum percentage of exposure to host-seeking mosquitoes that LLINs could have prevented for each individual (through personal protection) if they would have used the net while in bed.


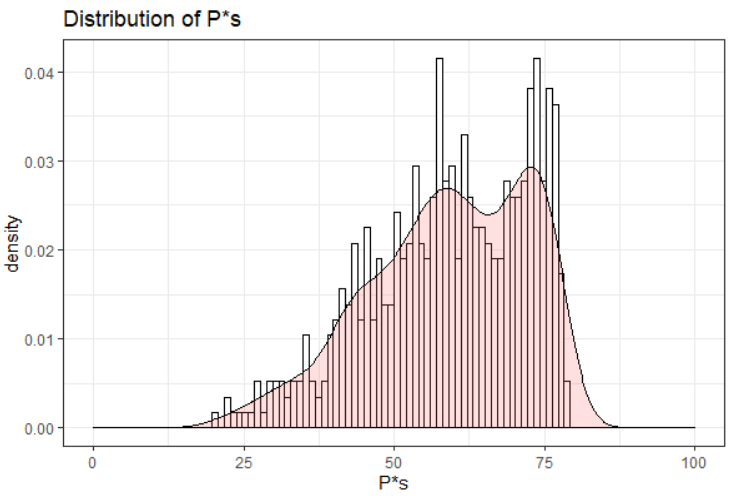

Supplement: S2 Fig — This is the maximum percentage of exposure to host-seeking mosquitoes that LLINs could have prevented for each individual (through personal protection) if they would have used the net while in bed. (DOCX) [file pone.0270882.s002.docx]
