# Supplementary material for: Overlaying human and mosquito behavioral data to estimate residual exposure to host-seeking mosquitoes and the protection of bednets in a malaria elimination setting where indoor residual spraying and nets were deployed together
Source: PLoS One. 2022 Sep 15;17(9):e0270882. doi: 10.1371/journal.pone.0270882 (PMC9477321; doi:10.1371/journal.pone.0270882)
Supplement: S3 Fig — Percentage of participants that were outdoors (grey area), indoors but not in bed (yellow), indoors in bed using an LLIN (green) and indoors in bed but not using an LLIN (red) during the low transmission (left panel) and high transmission season (right panel), including human behavioral data recorded after 8am. (DOCX) [file pone.0270882.s003.docx]

**S5 Fig. Location of study participants during the evening, night and morning.** Percentage of participants that were outdoors (grey area), indoors but not in bed (yellow), indoors in bed using an LLIN (green) and indoors in bed but not using an LLIN (red) during the low transmission (left panel) and high transmission season (right panel), including human behavioral data recorded after 8am.

| 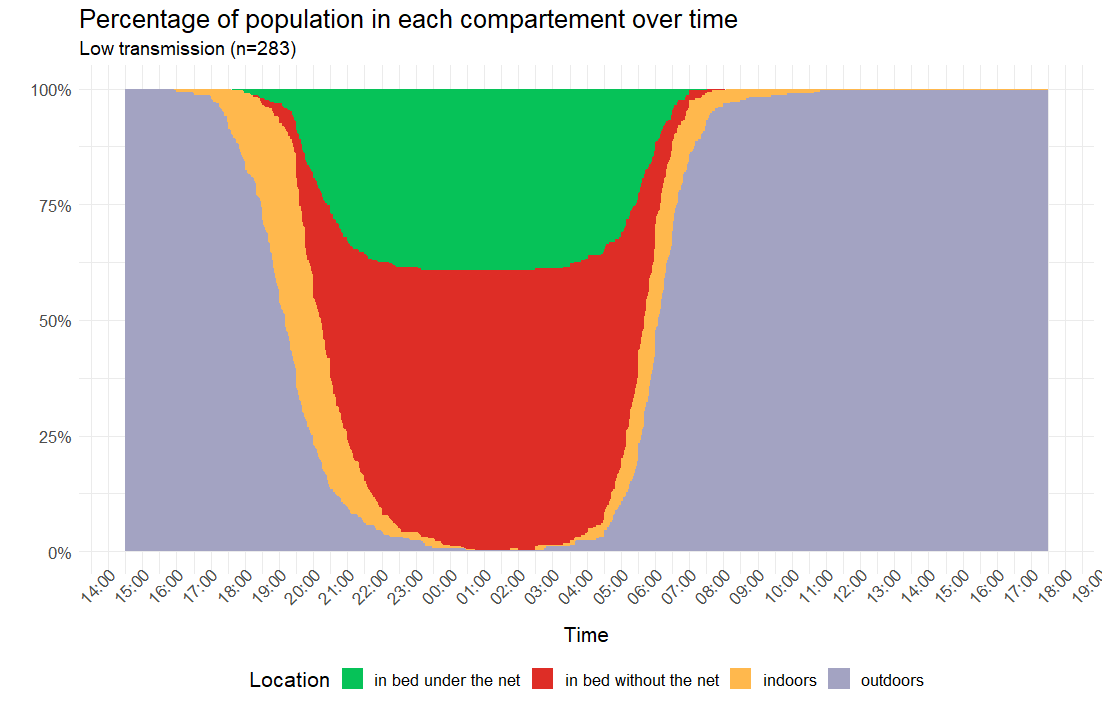 | 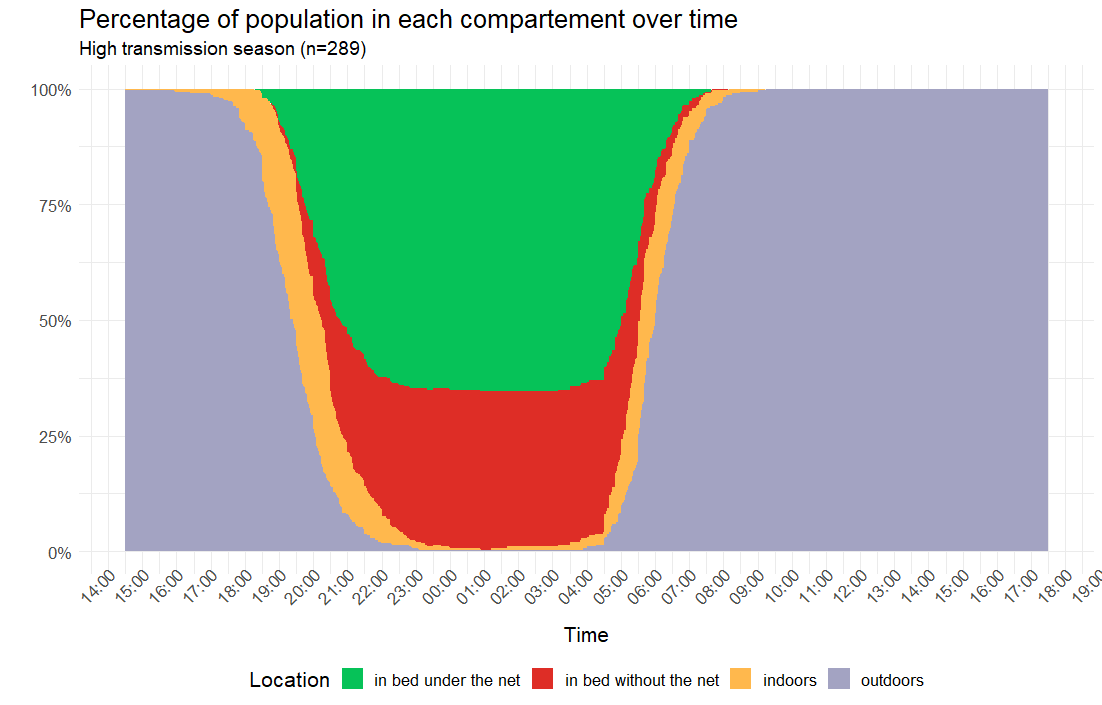 |
| --- | --- |

|  |  |
| --- | --- |
