# Supplementary material for: Overlaying human and mosquito behavioral data to estimate residual exposure to host-seeking mosquitoes and the protection of bednets in a malaria elimination setting where indoor residual spraying and nets were deployed together
Source: PLoS One. 2022 Sep 15;17(9):e0270882. doi: 10.1371/journal.pone.0270882 (PMC9477321; doi:10.1371/journal.pone.0270882)
Supplement: S1 Table — (DOCX) [file pone.0270882.s004.docx]

**S2 Table. Model parameters with their definitions and equations**

The following nomenclature is based on that proposed by [1]. In the original model, B indicates mosquito bites. In the present study, it refers to host-seeking mosquitoes.

| Notation | Definition | Calculation formula |
| --- | --- | --- |
| $B_{I, t}$ | Indoor biting rate at time t | $\frac{\sum_{t_{start}}^{t_{end}} {Host-seeking Mosquitos}_{indoors}}{People by Trap x Interval Minutes}$  Where the interval is the time interval in which time t is comprised and where  $t_{start}$: start of collection time interval (e.g. 18:00)  $t_{end}$: end of collection time interval (e.g. 20:00) |
| $B_{O, t}$ | Outdoor biting rate at time t | $\frac{\sum_{t_{start}}^{t_{end}} {Host-seeking Mosquitos}_{indoors}}{People by Trap x Interval Minutes}$  Where the interval is the time interval in which time t is comprised and where  $t_{start}$: start of collection time interval (e.g. 18:00)  $t_{end}$: end of collection time interval (e.g. 20:00) |
| $B_{O,bb}$ | Number of host-seeking mosquitoes per person outdoor (O) in the evening before going indoors (bb=before bed) during one night | $\sum_{t=18:00}^{t_{indoors}} B_{O,t}$ |
| $B_{I,bb}.$ | Number of host-seeking mosquitoes per person indoors (I) before going to bed (bb) during one night | $\sum_{t_{indoors}}^{t_{to bed}} B_{I,t}$ |
| $B_{I,bu}$ | Number of host-seeking mosquitoes per person indoors (I) while in bed unprotected (bu) during one night | $\sum_{t_{to bed}}^{t_{get up}} B_{I,t}$ |
| $B_{I,bp}$ | Number of host-seeking mosquitoes per person indoors (I) while in bed under the net (bp) during one night | $\rho\sum_{t_{to bed}}^{t_{get up}} B_{I,t}$  Where $\rho$ if the percentage of mosquitoes that successfully bite while people are under the net |
| $B_{I,ab}$ | Number of host-seeking mosquitoes per person indoors (I) after getting up from bed (ab) during one night | $\sum_{t_{get up}}^{t_{leave house}} B_{I,t}$ |
| $B_{O,ab}$ | Number of host-seeking mosquitoes per person outdoor (O) in the morning after getting up (ab=after bed) | $\sum_{t_{leave house}}^{t=08:00} B_{O,t}$ |
| $B_{I,b}$ | Number of host-seeking mosquitos per person indoors (I) while in bed (b) during one night | $\begin{matrix} if under net B_{I,bp} \\ if not under the net B_{I,bu} \end{matrix}$ |
| $B_{I}$ | Number of host-seeking mosquitos pr person indoors (I) during one night | $B_{I,bb}+ B_{I,b}+ .B_{I,ab}$ |
| $B_{O}$ | Number of host-seking mosquitoes per person outdoors (O) during one night | $B_{O,bb}+ .B_{O,ab}$ |
| $B$ | Number of host-seeking mosquitoes per person indoors and outdoors during one night at observed levels of bednet use | $B_{O}+ B_{I}={B_{O,bb}+ B}_{I,bb}+ B_{I,b}+ .B_{I,ab}+B_{O,ab}$  $B_{I,b}$ for each participants is calculated as $B_{I,bp}$ or $B_{I,bu}$ depending on whether the participant used the net to sleep |
| $B_{u}$ | Number of host-seeking mosquitoes person during on night if not sleeping under the net (  u=unprotected) | ${B_{O,bb}+ B}_{I,bb}+ B_{I,bu}+ .B_{I,ab}+B_{O,ab}$ |
| $B_{p}$ | Number of host-seeking mosquitoes received by a person during on night if sleeping under the net (p=protected) | ${B_{O,bb}+ B}_{I,bb}+ B_{I,bp}+ .B_{I,ab}+B_{O,ab}$ |
| $B_{I,nb}$ | Number of host-seeking mosquitoes per person while indoors (I) but not in bed (nb) during one night | $B_{I,bb}+ B_{I,ab}$ |
| $B_{I,p}$ | Number of host-seeking mosquitoes per person indoors (I) if sleeping under the net (p=protected) during one night | $B_{I,bb}+ B_{I,bp}+B_{I,ab}$ |
| $B_{I,u}$ | Number of host-seeking mosquitoes per person indoors (I) if not sleeping under the net (u=unprotect) during one night | $B_{I,bb}+ B_{I,bu}+B_{I,ab}$ |
| $B_{r}$ | Total number of residual host-seeking mosquitoes (r) that all participants together were exposed to during one night given the reported levels of bed net use | $\sum_{p=1}^{p=n} B$  Where p=participant and n=max number of participants in the study and $B_{I,b}$ for each participants is calculated as $B_{I,bp}$ or $B_{I,bu}$ depending on whether the participant used the net to sleep or not |
| $B_{rp}$ | Total number of residual host-seeking mosquitoes (r) that all participants together were exposed to during one night if they would have all used a net to sleep (p=protected) | $\sum_{p=1}^{p=n} B_{p}$  Where p=participant and n=max number of participants in the study and where $B_{I,b}=B_{I,bp}$ |
| $B_{ru}$ | Total number of host-seeking mosquitoes (r) that all participants together would have been exposed to during on night if none of them would have used a net to sleep (u=unprotected) | $\sum_{p=1}^{p=n} B_{u}$  Where p=participant and n=max number of participants in the study and where $B_{I,b}=B_{I,bu}$ |
| $\pi_{O,bb}$ | Percentage of host-seeking mosquitoes that a person was exposed tooutdoors (O) before going indoors (bb) during one night | $100x\frac{B_{O,bb}}{B}$ |
| $\pi_{I,bb}$ | Percentage of host-seeking mosquitoes that a person was exposed to indoors (I) before going to bed (bb) during one night | $100x\frac{B_{I,bb}}{B}$ |
| $\pi_{I,b}$ | Percentage of host-seeking mosquitoes that a person was exposed indoors (I) while in bed (b) during one night | $100x\frac{B_{I,b}}{B}$ |
| $\pi_{I,ab}$ | Percentage of host-seeking mosquitoes that a person was exposed indoors (I) after getting up during (ab=after bed) one night | $100x\frac{B_{I,ab}}{B}$ |
| $\pi_{O,ab}$ | Percentage of host-seeking mosquitoes that a person was exposed outdoors (O) after getting up (ab= after bed) during one night | $100x\frac{B_{O,ab}}{B}$ |
| $\pi_{Op,bb}$ | Percentage of host-seeking mosquitoes that a person would have been exposed to indoors (I) before going to bed (bb) during one night, assuming bednet use | $100x\frac{B_{O,bb}}{B_{p}}$ |
| $\pi_{Ip,bb}$ | Percentage of host-seeking mosquitoes that a person would have been exposed to indoors (I) before going to bed (bb) during one night, assuming bednet use | $100x\frac{B_{I,bb}}{B_{p}}$ |
| $\pi_{Ip,b}$ | Percentage of host-seeking mosquitoes that a person would have been exposed to indoors (I) while in bed (b) during one night, assuming bednet use | $100x\frac{B_{I,b}}{B_{p}}$ |
| $\pi_{Ip,ab}$ | Percentage of host-seeking mosquitoes that a person would have been exposed to indoors (I) after getting up (ab=after bed) during one night, assuming bednet use | $100x\frac{B_{I,ab}}{B_{p}}$ |
| $\pi_{Op,ab}$ | Percentage of host-seeking mosquitoes that a person would have been exposed tooutdoors (O) after getting up (ab=after bed) during one night, assuming bednet use | $100x\frac{B_{O,ab}}{B_{p}}$ |
| $\pi_{low}$ | Percentage of residual host-seeking mosquitoes that participants were exposed to during the low transmission season (low) | $\frac{\sum_{p=1}^{p=n (low trasmision)} B}{\sum_{p=1}^{p=n (high trasmision)} B+ \sum_{p=1}^{p=n (low trasmision)} B}$ |
| $\pi_{p,low}$ | Percentage of host-seeking mosquitoes that participants would have been exposed to(p=protected) in the low transmission season if all participants would have used anet to sleep | $\frac{\sum_{p=1}^{p=n (low trasmision)} B_{p}}{\sum_{p=1}^{p=n (high trasmision)} B_{p}+ \sum_{p=1}^{p=n (low trasmision)} B_{p}}$ |
| $P_{S,C}^{*}$ | Percentage of exposure to host-seeking mosquitoes that LLIN prevented at observed levels of bednet use | $100x(1- \frac{\sum_{p=1}^{p=n} B}{\sum_{p=1}^{p=n} B_{u}})$ |
| $P_{S}^{*}$ | Percentage of exposure to host-seeking mosquitoes that LLINs could have prevented if all participants would have used a net to sleep | $100x(1- \frac{B_{p}}{B_{u}})$ |

1. Killeen GF, Kihonda J, Lyimo E, Oketch FR, Kotas ME, Mathenge E, et al. Quantifying behavioural interactions between humans and mosquitoes: Evaluating the protective efficacy of insecticidal nets against malaria transmission in rural Tanzania. BMC Infect Dis. 2006;6: 1–10. doi:10.1186/1471-2334-6-161
