# Supplementary material for: Overlaying human and mosquito behavioral data to estimate residual exposure to host-seeking mosquitoes and the protection of bednets in a malaria elimination setting where indoor residual spraying and nets were deployed together
Source: PLoS One. 2022 Sep 15;17(9):e0270882. doi: 10.1371/journal.pone.0270882 (PMC9477321; doi:10.1371/journal.pone.0270882)
Supplement: S1 Box — (DOCX) [file pone.0270882.s005.docx]

**S3 Box: Exclusions criteria for mosquito surveillance data**

| **Mosquito collections meeting the following criteria were excluded from the analyses.**   - The fan and/or light of the trap was not working; - Collection bottle was not properly attached; - Synthetic lure and/or artificial CO2 source not placed/connected properly. - The household resident(s) did not sleep under their LLIN next to the trap, - More than two people slept next to the trap; - Ants found in a collection bottle; - The bottle rotator had not completed all programmed rotations; - Collections started before 4pm or extended beyond 8am. |
| --- |
