# Supplementary material for: Overlaying human and mosquito behavioral data to estimate residual exposure to host-seeking mosquitoes and the protection of bednets in a malaria elimination setting where indoor residual spraying and nets were deployed together
Source: PLoS One. 2022 Sep 15;17(9):e0270882. doi: 10.1371/journal.pone.0270882 (PMC9477321; doi:10.1371/journal.pone.0270882)
Supplement: S1 File — (DOCX) [file pone.0270882.s006.docx]

**S6 Structured questionnaire to assess human behaviors***.*

*Original Version (in Portuguese)*

|  | **Variable / Field Name** | **Field Label**  ***Field Note*** | **Field Attributes (Field Type, Validation, Choices, Calculations, etc.)** |
| --- | --- | --- | --- |
| 1 | uri | URI | text |
| 2 | geopoint_lat | 2.Coordinates_latitude | text |
| 3 | geopoint_lng | 2.Coordinates_longitude | text |
| 4 | geopoint_acc | 2.Coordinates_precisao | text |
| 5 | geopoint_alt | 2.Coordinates_altitude | text |
| 6 | start_date | Data | text (date_dmy) |
| 7 | posto | 3. Posto Administrativo | text |
| 8 | localidade | 3.1. Localidade | text |
| 9 | perm_id | 4. Número do Perm ID do entrevistado | text |
| 10 | name | 5. Nome do entrevistado | text, Required |
| 11 | p_mainlist | 6. A pessoa entrevistada pertence a lista principal | radio   \| 1 \| Sim \| \| --- \| --- \| \| 2 \| Nao \| |
| 12 | p_present | 7. A pessoa entrevistada está presente? | radio, Required   \| 1 \| Sim \| \| --- \| --- \| \| 2 \| Nao \| |
| 13 | p_reason_present | 8. Se não, porquê não está? | radio   \| 1 \| A pessoa imigrou para outro local \| \| --- \| --- \| \| 2 \| Ele não estava em casa no momento da entrevista \| \| 3 \| Eles rejeitaram a participação \| \| 4 \| A pessoa morreu \| \| 5 \| Nunca houve uma pessoa com esse nome nesta casa \| \| 6 \| A pessoa não é da idade correspondida \| \| 7 \| Não esta, está trabalhar fora \| \| 8 \| O permID nao pertence a esta pessoa \| |
| 14 | age | 9. Qual é a idade? | text (integer) |
| 15 | related_qes | 10. As questões respondidas são relacionadas a? | radio   \| 1 \| Criança entre 5 e 11 anos \| \| --- \| --- \| \| 2 \| Crianças entre 12 e 17 anos \| \| 3 \| Adulto, o qual deve ser o entrevistado \| |
| 16 | p_work | 11. Você trabalha de noite até amanhecer (pode ser meio-tempo)? | radio   \| 1 \| Sim, trabalho formal \| \| --- \| --- \| \| 2 \| Sim, trabalho por conta propria \| \| 3 \| Não \| \| 4 \| Não quero dizer \| |
| 17 | work_type | 12. Por favor, especifique tipo de trabalho | text |
| 18 | nigth_work | 13. Se sim, voce faz sempre turno noturno? | radio   \| 1 \| Sim \| \| --- \| --- \| \| 2 \| Não, tenho alternativa de turnos de dia e de noite \| \| 3 \| Somente ontem \| \| 4 \| Outros(especifique) \| |
| 19 | oth_nigth_work | 13.1. Outros(especifique) | text |
| 20 | freq_nigth_work | 14. Durante quantos dias de semana voce trabalha a noite? | radio   \| 1 \| 1 dia \| \| --- \| --- \| \| 2 \| 2 dias \| \| 3 \| 3 dias \| \| 4 \| 4 dias \| \| 5 \| 5 dias \| \| 6 \| 6 dias \| \| 7 \| 7 dias \| \| 8 \| 0 dias \| \| 9 \| Não sei \| |
| 21 | p_respondent | 15. Quem está a responder as questões? | radio   \| 1 \| Pai/Mãe \| \| --- \| --- \| \| 2 \| Irmão/Irmã \| \| 3 \| Avó/Avô \| \| 4 \| A propia criança que deve ser entrevistada \| \| 5 \| Outro adulto cuidador da criança \| |
| 22 | rain | 16. Choveu na noite passada? | radio   \| 1 \| Sim \| \| --- \| --- \| \| 2 \| Não \| \| 3 \| Não sei \| |
| 23 | change_plans | 17. Se sim, mudou os planos de actividades para esse dia? | radio   \| 1 \| Sim \| \| --- \| --- \| \| 2 \| Não \| \| 3 \| Não sei \| |
| 24 | place_sleep | 18. Onde é que dormiu na noite passada? | radio   \| 1 \| Dentro de casa \| \| --- \| --- \| \| 2 \| Fora de casa \| \| 3 \| Dormi no quintal \| \| 4 \| Não dormi \| |
| 25 | sleep_bednet | 19. Você ou a criança dormiram em baixo da rede mosquiteira? | radio   \| 1 \| Sim \| \| --- \| --- \| \| 2 \| Não \| \| 3 \| Recusa responder \| |
| 26 | yard_before | 20. Ao anoitecer, antes de ir para cama, você ou a criança estiveram durante algum tempo no quintal? | radio   \| 1 \| Sim \| \| --- \| --- \| \| 2 \| Não \| \| 3 \| Não sei \| |
| 27 | actitivities | 21. Quais foram as actividades? | checkbox   \| 1 \| actitivities___1 \| Cânticos diversos \| \| --- \| --- \| --- \| \| 2 \| actitivities___2 \| Somente sentado \| \| 3 \| actitivities___3 \| Limpeza \| \| 4 \| actitivities___4 \| Cozinhar \| \| 5 \| actitivities___5 \| Banho \| \| 6 \| actitivities___6 \| Jogos \| \| 7 \| actitivities___7 \| Exercícios físicos/desportos \| \| 8 \| actitivities___8 \| Socializar (conversar, beber) \| \| 9 \| actitivities___9 \| Ver televisão \| \| 10 \| actitivities___10 \| Ouvir rádio \| \| 11 \| actitivities___11 \| Alimentar ou dar banho a criança/bebe \| \| 12 \| actitivities___12 \| Estudar \| \| 13 \| actitivities___13 \| Trabalhar \| \| 14 \| actitivities___14 \| Outros especificar \| |
| 28 | oth_actitivities | Outros, especificar | text |
| 29 | act_place1 | Section Header: *22. Indica para cada actividade seleccionada se foi realizada num local fechado e/ou aberto (ambas podem ser possíveis).*  Cânticos diversos | radio (Matrix)   \| 1 \| Num lugar fechado \| \| --- \| --- \| \| 2 \| Ao ar livre \| \| 3 \| Ambas \| |
| 30 | act_place2 | Somente sentado | radio (Matrix)   \| 1 \| Num lugar fechado \| \| --- \| --- \| \| 2 \| Ao ar livre \| \| 3 \| Ambas \| |
| 31 | act_place3 | Limpeza | radio (Matrix)   \| 1 \| Num lugar fechado \| \| --- \| --- \| \| 2 \| Ao ar livre \| \| 3 \| Ambas \| |
| 32 | act_place4 | Cozinhar | radio (Matrix)   \| 1 \| Num lugar fechado \| \| --- \| --- \| \| 2 \| Ao ar livre \| \| 3 \| Ambas \| |
| 33 | act_place5 | Banho | radio (Matrix)   \| 1 \| Num lugar fechado \| \| --- \| --- \| \| 2 \| Ao ar livre \| \| 3 \| Ambas \| |
| 34 | act_place6 | Jogos | radio (Matrix)   \| 1 \| Num lugar fechado \| \| --- \| --- \| \| 2 \| Ao ar livre \| \| 3 \| Ambas \| |
| 35 | act_place7 | Exercícios físicos/desportos | radio (Matrix)   \| 1 \| Num lugar fechado \| \| --- \| --- \| \| 2 \| Ao ar livre \| \| 3 \| Ambas \| |
| 36 | act_place8 | Socializar (conversar, beber) | radio (Matrix)   \| 1 \| Num lugar fechado \| \| --- \| --- \| \| 2 \| Ao ar livre \| \| 3 \| Ambas \| |
| 37 | act_place9 | Ver televisão | radio (Matrix)   \| 1 \| Num lugar fechado \| \| --- \| --- \| \| 2 \| Ao ar livre \| \| 3 \| Ambas \| |
| 38 | act_place10 | Ouvir rádio | radio (Matrix)   \| 1 \| Num lugar fechado \| \| --- \| --- \| \| 2 \| Ao ar livre \| \| 3 \| Ambas \| |
| 39 | act_place11 | Alimentar ou dar banho a criança/bebe | radio (Matrix)   \| 1 \| Num lugar fechado \| \| --- \| --- \| \| 2 \| Ao ar livre \| \| 3 \| Ambas \| |
| 40 | act_place12 | Estudar | radio (Matrix)   \| 1 \| Num lugar fechado \| \| --- \| --- \| \| 2 \| Ao ar livre \| \| 3 \| Ambas \| |
| 41 | act_place13 | Trabalhar | radio (Matrix)   \| 1 \| Num lugar fechado \| \| --- \| --- \| \| 2 \| Ao ar livre \| \| 3 \| Ambas \| |
| 42 | act_place14 | Outros(a actividade que voce ja escreveu) | radio (Matrix)   \| 1 \| Num lugar fechado \| \| --- \| --- \| \| 2 \| Ao ar livre \| \| 3 \| Ambas \| |
| 43 | time_act | 23. Qual actividade levou mais tempo | radio   \| 1 \| Cânticos diversos \| \| --- \| --- \| \| 2 \| Somente sentado \| \| 3 \| Limpeza \| \| 4 \| Cozinhar \| \| 5 \| Banho \| \| 6 \| Jogos \| \| 7 \| Exercícios físicos/desportos \| \| 8 \| Socializar (conversar, beber) \| \| 9 \| Ver televisão \| \| 10 \| Ouvir rádio \| \| 11 \| Alimentar ou dar banho a criança/bebe \| \| 12 \| Estudar \| \| 13 \| Trabalhar \| \| 14 \| Outros, especificar \| |
| 44 | prevent_mosquitos | 24. Você ou a criança usaram algum produto para prevenir picadas de mosquitos DURANTE O DESEMPENHO DE ESSAS ACTIVIDADES?? | checkbox   \| 1 \| prevent_mosquitos___1 \| Spray com insecticida (Baygon) \| \| --- \| --- \| --- \| \| 2 \| prevent_mosquitos___2 \| Carvão anti-mosquito (Dragão) \| \| 3 \| prevent_mosquitos___3 \| Repelente \| \| 4 \| prevent_mosquitos___4 \| Fumo (sulé) \| \| 5 \| prevent_mosquitos___5 \| Roupa \| \| 6 \| prevent_mosquitos___6 \| Rede Mosquiteira \| \| 7 \| prevent_mosquitos___7 \| Nenhum \| \| 8 \| prevent_mosquitos___8 \| Outros, especificar \| |
| 45 | oth_prevent_mosquitos | 24.1. Outros, especificar | text |
| 46 | out_yard | 25. Ao anoitecer, antes de ir a cama, você ou a criança por algum momento estiveram fora do quintal? | radio   \| 1 \| Sim \| \| --- \| --- \| \| 2 \| Não \| \| 3 \| Não sei \| |
| 47 | where_yard | 26. Onde você esteve? | checkbox   \| 1 \| where_yard___1 \| Trabalhando \| \| --- \| --- \| --- \| \| 2 \| where_yard___2 \| Na machamba \| \| 3 \| where_yard___3 \| Na casa de parentes ou vizinhos (conversando, cozinhando, outros..) \| \| 4 \| where_yard___4 \| Barraca, discoteca, clube \| \| 5 \| where_yard___5 \| Restaurante \| \| 6 \| where_yard___6 \| Igreja \| \| 7 \| where_yard___7 \| Shoping \| \| 8 \| where_yard___8 \| Mercado \| \| 9 \| where_yard___9 \| Reunião/Encontro com lider comunitário/casa do secretário do bairro \| \| 10 \| where_yard___10 \| Funeral \| \| 11 \| where_yard___11 \| Hospital \| \| 12 \| where_yard___12 \| Unidade Sanitaria \| \| 13 \| where_yard___13 \| Passear \| \| 14 \| where_yard___14 \| Outro, especificar \| |
| 48 | oth_where_yard | 26.1. Outro, especificar | text |
| 49 | oth_place1 | Section Header: *27. Indica para cada actividade seleccionada se foi realizada num local fechado e/ou aberto (ambas podem ser possíveis).*  Trabalhando | radio (Matrix)   \| 1 \| Num lugar fechado \| \| --- \| --- \| \| 2 \| Ao ar livre \| \| 3 \| Ambas \| |
| 50 | oth_place2 | Na machamba | radio (Matrix)   \| 1 \| Num lugar fechado \| \| --- \| --- \| \| 2 \| Ao ar livre \| \| 3 \| Ambas \| |
| 51 | oth_place3 | Na casa de parentes ou vizinhos (conversando, cozinhando, outros..) | radio (Matrix)   \| 1 \| Num lugar fechado \| \| --- \| --- \| \| 2 \| Ao ar livre \| \| 3 \| Ambas \| |
| 52 | oth_place4 | Barraca, discoteca, clube | radio (Matrix)   \| 1 \| Num lugar fechado \| \| --- \| --- \| \| 2 \| Ao ar livre \| \| 3 \| Ambas \| |
| 53 | oth_place5 | Restaurante | radio (Matrix)   \| 1 \| Num lugar fechado \| \| --- \| --- \| \| 2 \| Ao ar livre \| \| 3 \| Ambas \| |
| 54 | oth_place6 | Igreja | radio (Matrix)   \| 1 \| Num lugar fechado \| \| --- \| --- \| \| 2 \| Ao ar livre \| \| 3 \| Ambas \| |
| 55 | oth_place7 | Shoping | radio (Matrix)   \| 1 \| Num lugar fechado \| \| --- \| --- \| \| 2 \| Ao ar livre \| \| 3 \| Ambas \| |
| 56 | oth_place8 | Mercado | radio (Matrix)   \| 1 \| Num lugar fechado \| \| --- \| --- \| \| 2 \| Ao ar livre \| \| 3 \| Ambas \| |
| 57 | oth_place9 | Reunião/Encontro com lider comunitário/casa do secretário do bairro | radio (Matrix)   \| 1 \| Num lugar fechado \| \| --- \| --- \| \| 2 \| Ao ar livre \| \| 3 \| Ambas \| |
| 58 | oth_place10 | Funeral | radio (Matrix)   \| 1 \| Num lugar fechado \| \| --- \| --- \| \| 2 \| Ao ar livre \| \| 3 \| Ambas \| |
| 59 | oth_place11 | Hospital | radio (Matrix)   \| 1 \| Num lugar fechado \| \| --- \| --- \| \| 2 \| Ao ar livre \| \| 3 \| Ambas \| |
| 60 | oth_place12 | Unidade Sanitaria | radio (Matrix)   \| 1 \| Num lugar fechado \| \| --- \| --- \| \| 2 \| Ao ar livre \| \| 3 \| Ambas \| |
| 61 | oth_place13 | Passear | radio (Matrix)   \| 1 \| Num lugar fechado \| \| --- \| --- \| \| 2 \| Ao ar livre \| \| 3 \| Ambas \| |
| 62 | oth_place14 | Outro, especificar | radio (Matrix)   \| 1 \| Num lugar fechado \| \| --- \| --- \| \| 2 \| Ao ar livre \| \| 3 \| Ambas \| |
| 63 | spend_time_place | 28. Onde você ou a criança passaram a maior parte do vosso tempo | radio   \| 1 \| Trabalhando \| \| --- \| --- \| \| 2 \| Na machamba \| \| 3 \| Na casa de parentes ou vizinhos (conversando, cozinhando, outros..) \| \| 4 \| Barraca, discoteca, clube \| \| 5 \| Restaurante \| \| 6 \| Igreja \| \| 7 \| Shoping \| \| 8 \| Mercado \| \| 9 \| Reunião/Encontro com lider comunitário/casa do secretário do bairro \| \| 10 \| Funeral \| \| 11 \| Hospital \| \| 12 \| Unidade Sanitaria \| \| 13 \| Passear \| \| 14 \| Outro, especificar \| |
| 64 | prev_mosq | 29. Você ou a criança usaram algum produto para prevenir picadas de mosquitos DURANTE O DESEMPENHO DE ESSAS ACTIVIDADES? | checkbox, Required   \| 1 \| prev_mosq___1 \| Spray com insecticida (Baygon) \| \| --- \| --- \| --- \| \| 2 \| prev_mosq___2 \| Carvão anti-mosquito (Dragão) \| \| 3 \| prev_mosq___3 \| Repelente \| \| 4 \| prev_mosq___4 \| Fumo (sulé) \| \| 5 \| prev_mosq___5 \| Roupa \| \| 6 \| prev_mosq___6 \| Rede Mosquiteira \| \| 7 \| prev_mosq___7 \| Nenhum \| \| 8 \| prev_mosq___8 \| Outros, especificar \| |
| 65 | oth_prev_mosq | 29.1. Outros, especificar | text |
| 66 | weekend_evening | Durante a tarde-noite, voçe faz actividades distintas no final de semana do que durante a semana? | radio   \| 1 \| Sim \| \| --- \| --- \| \| 2 \| Nao \| |
| 67 | out_sleep | 30. Você ou a criança acordaram e sairam para fora da cama/esteira durante a hora de dormir? | radio   \| 1 \| Sim \| \| --- \| --- \| \| 2 \| Não \| \| 3 \| Não sei \| |
| 68 | reason_out_sleep | 31. Se sim, qual foi o motivo? | checkbox   \| 1 \| reason_out_sleep___1 \| Recebi uma visita inesperada \| \| --- \| --- \| --- \| \| 2 \| reason_out_sleep___2 \| Tive que cuidar dos animais \| \| 3 \| reason_out_sleep___3 \| Ouvi um barulho e fui verificar \| \| 4 \| reason_out_sleep___4 \| Fui a casa de banho \| \| 5 \| reason_out_sleep___5 \| Acordei por causa do bebé \| \| 6 \| reason_out_sleep___6 \| Não sei \| \| 7 \| reason_out_sleep___7 \| Outros, especificar \| |
| 69 | oth_reason_out_sleep | 31.1. Outro, especificar | text |
| 70 | night_location_toilet | 31.2 Voçe saiu de casa para ir a casa de banho? | checkbox   \| 1 \| night_location_toilet___1 \| Sim, foi a minha casa de banho \| \| --- \| --- \| --- \| \| 2 \| night_location_toilet___2 \| Sim, foi ao campo \| \| 3 \| night_location_toilet___3 \| Não, fiz no balde no meu quarto \| |
| 71 | place_wc | 32. Onde fica a casa de banho? | radio   \| 1 \| Fora da casa \| \| --- \| --- \| \| 2 \| Dentro da casa \| \| 3 \| Noutra casa/vizinho \| \| 4 \| Não tenho casa de banho \| \| 5 \| Não quero responder \| \| 6 \| Céu aberto (no mato). \| |
| 72 | sleep_prod_prev | 33. Você ou a criança usaram algum produto para prevenir picadas de mosquitos DURANTE O DESEMPENHO DE ESSAS ACTIVIDADES? | checkbox   \| 1 \| sleep_prod_prev___1 \| Spray com insecticida (Baygon) \| \| --- \| --- \| --- \| \| 2 \| sleep_prod_prev___2 \| Carvão anti-mosquito (Dragão) \| \| 3 \| sleep_prod_prev___3 \| Repelente \| \| 4 \| sleep_prod_prev___4 \| Fumo (sulé) \| \| 5 \| sleep_prod_prev___5 \| Roupa \| \| 6 \| sleep_prod_prev___6 \| Rede Mosquiteira \| \| 7 \| sleep_prod_prev___7 \| Nenhum \| \| 8 \| sleep_prod_prev___8 \| Outros, especificar \| |
| 73 | oth_sleep_prod_prev | 33.1. Outro, especificar | text |
| 74 | sun | 34. Quando você acordou já tinha sol (nascer-do-sol)? | radio   \| 1 \| Sim \| \| --- \| --- \| \| 2 \| Não \| |
| 75 | wake_up_yard | 35. Depois de acordar, antes de sair o sol você ou a criança estiveram durante algum tempo no quintal? | radio   \| 1 \| Sim \| \| --- \| --- \| \| 2 \| Não \| \| 3 \| Não sei \| |
| 76 | wake_actv | 36. Quais foram as actividades? | checkbox   \| 1 \| wake_actv___1 \| Cânticos diversos \| \| --- \| --- \| --- \| \| 2 \| wake_actv___2 \| Somente sentado \| \| 3 \| wake_actv___3 \| Limpeza \| \| 4 \| wake_actv___4 \| Cozinhar \| \| 5 \| wake_actv___5 \| Banho \| \| 6 \| wake_actv___6 \| Jogos \| \| 7 \| wake_actv___7 \| Exercícios físicos/desportos \| \| 8 \| wake_actv___8 \| Socializar (conversar, beber) \| \| 9 \| wake_actv___9 \| Ver televisão \| \| 10 \| wake_actv___10 \| Ouvir rádio \| \| 11 \| wake_actv___11 \| Alimentar ou dar banho a criança/bebe \| \| 12 \| wake_actv___12 \| Estudar \| \| 13 \| wake_actv___13 \| Trabalhar \| \| 14 \| wake_actv___14 \| Outros, especificar \| |
| 77 | oth_wake_actv | 36.1. Outro, especificar | text |
| 78 | wk_place1 | Section Header: *37. Indica para cada actividade seleccionada se foi realizada num local fechado e/ou aberto (ambas podem ser possíveis).*  Cânticos diversos | radio (Matrix)   \| 1 \| Num lugar fechado \| \| --- \| --- \| \| 2 \| Ao ar livre \| \| 3 \| Ambas \| |
| 79 | wk_place2 | Somente sentado | radio (Matrix)   \| 1 \| Num lugar fechado \| \| --- \| --- \| \| 2 \| Ao ar livre \| \| 3 \| Ambas \| |
| 80 | wk_place3 | Limpeza | radio (Matrix)   \| 1 \| Num lugar fechado \| \| --- \| --- \| \| 2 \| Ao ar livre \| \| 3 \| Ambas \| |
| 81 | wk_place4 | Cozinhar | radio (Matrix)   \| 1 \| Num lugar fechado \| \| --- \| --- \| \| 2 \| Ao ar livre \| \| 3 \| Ambas \| |
| 82 | wk_place5 | Banho | radio (Matrix)   \| 1 \| Num lugar fechado \| \| --- \| --- \| \| 2 \| Ao ar livre \| \| 3 \| Ambas \| |
| 83 | wk_place6 | Jogos | radio (Matrix)   \| 1 \| Num lugar fechado \| \| --- \| --- \| \| 2 \| Ao ar livre \| \| 3 \| Ambas \| |
| 84 | wk_place7 | Exercícios físicos/desportos | radio (Matrix)   \| 1 \| Num lugar fechado \| \| --- \| --- \| \| 2 \| Ao ar livre \| \| 3 \| Ambas \| |
| 85 | wk_place8 | Socializar (conversar, beber) | radio (Matrix)   \| 1 \| Num lugar fechado \| \| --- \| --- \| \| 2 \| Ao ar livre \| \| 3 \| Ambas \| |
| 86 | wk_place9 | Ver televisão | radio (Matrix)   \| 1 \| Num lugar fechado \| \| --- \| --- \| \| 2 \| Ao ar livre \| \| 3 \| Ambas \| |
| 87 | wk_place10 | Ouvir rádio | radio (Matrix)   \| 1 \| Num lugar fechado \| \| --- \| --- \| \| 2 \| Ao ar livre \| \| 3 \| Ambas \| |
| 88 | wk_place11 | Alimentar our dar banho a criança/bebe | radio (Matrix)   \| 1 \| Num lugar fechado \| \| --- \| --- \| \| 2 \| Ao ar livre \| \| 3 \| Ambas \| |
| 89 | wk_place12 | Estudar | radio (Matrix)   \| 1 \| Num lugar fechado \| \| --- \| --- \| \| 2 \| Ao ar livre \| \| 3 \| Ambas \| |
| 90 | wk_place13 | Trabalhar | radio (Matrix)   \| 1 \| Num lugar fechado \| \| --- \| --- \| \| 2 \| Ao ar livre \| \| 3 \| Ambas \| |
| 91 | wk_place14 | Outra, especificar | radio (Matrix)   \| 1 \| Num lugar fechado \| \| --- \| --- \| \| 2 \| Ao ar livre \| \| 3 \| Ambas \| |
| 92 | wake_act_time | 38. Qual actividade levou mais tempo? | radio   \| 1 \| Cânticos diversos \| \| --- \| --- \| \| 2 \| Somente sentado \| \| 3 \| Limpeza \| \| 4 \| Cozinhar \| \| 5 \| Banho \| \| 6 \| Jogos \| \| 7 \| Exercícios físicos/desportos \| \| 8 \| Socializar (conversar, beber) \| \| 9 \| Ver televisão \| \| 10 \| Ouvir rádio \| \| 11 \| Alimentar ou dar banho a criança/bebe \| \| 12 \| Estudar \| \| 13 \| Trabalhar \| \| 14 \| Outros, especificar \| |
| 93 | wake_prod_prev | 39. Você ou a criança usaram algum produto para prevenir picadas de mosquitos DURANTE O DESEMPENHO DE ESSAS ACTIVIDADES? | checkbox   \| 1 \| wake_prod_prev___1 \| Spray com insecticida (Baygon) \| \| --- \| --- \| --- \| \| 2 \| wake_prod_prev___2 \| Carvão anti-mosquito (Dragão) \| \| 3 \| wake_prod_prev___3 \| Repelente \| \| 4 \| wake_prod_prev___4 \| Fumo (sulé) \| \| 5 \| wake_prod_prev___5 \| Roupa \| \| 6 \| wake_prod_prev___6 \| Rede Mosquiteira \| \| 7 \| wake_prod_prev___7 \| Nenhum \| \| 8 \| wake_prod_prev___8 \| Outros, especificar \| |
| 94 | oth_wake_prod_prev | 39.1. Outro, especificar | text |
| 95 | out_afterwk | 40. Depois de acordar, antes de sair o sol, você ou a criança estiveram por algum momento fora do quintal? | radio   \| 1 \| Sim \| \| --- \| --- \| \| 2 \| Não \| \| 3 \| Não sei \| |
| 96 | location_afterwk | 41. Onde você esteve? | checkbox   \| 1 \| location_afterwk___1 \| Trabalhando \| \| --- \| --- \| --- \| \| 2 \| location_afterwk___2 \| Na machamba \| \| 3 \| location_afterwk___3 \| Na casa de parentes ou vizinhos (conversando, cozinhando, outros..) \| \| 4 \| location_afterwk___4 \| Barraca, discoteca, clube \| \| 5 \| location_afterwk___5 \| Restaurante \| \| 6 \| location_afterwk___6 \| Igreja \| \| 7 \| location_afterwk___7 \| Shoping \| \| 8 \| location_afterwk___8 \| Mercado \| \| 9 \| location_afterwk___9 \| Reunião/Encontro com lider comunitário/casa do secretário do bairro \| \| 10 \| location_afterwk___10 \| Funeral \| \| 11 \| location_afterwk___11 \| Hospital \| \| 12 \| location_afterwk___12 \| Unidade Sanitaria \| \| 13 \| location_afterwk___13 \| Passear \| \| 14 \| location_afterwk___14 \| Outro, especificar \| |
| 97 | oth_location_afterwk | 41.1. Outro, especificar | text |
| 98 | afterwk_place1 | Section Header: *42. Indica para cada actividade seleccionada se foi realizada num local fechado e/ou aberto (ambas podem ser possíveis).*  Trabalhando | radio (Matrix)   \| 1 \| Num lugar fechado \| \| --- \| --- \| \| 2 \| Ao ar livre \| \| 3 \| Ambas \| |
| 99 | afterwk_place2 | Na machamba | radio (Matrix)   \| 1 \| Num lugar fechado \| \| --- \| --- \| \| 2 \| Ao ar livre \| \| 3 \| Ambas \| |
| 100 | afterwk_place3 | Na casa de parentes ou vizinhos (conversando, cozinhando, outros..) | radio (Matrix)   \| 1 \| Num lugar fechado \| \| --- \| --- \| \| 2 \| Ao ar livre \| \| 3 \| Ambas \| |
| 101 | afterwk_place4 | Barraca, discoteca, clube | radio (Matrix)   \| 1 \| Num lugar fechado \| \| --- \| --- \| \| 2 \| Ao ar livre \| \| 3 \| Ambas \| |
| 102 | afterwk_place5 | Restaurante | radio (Matrix)   \| 1 \| Num lugar fechado \| \| --- \| --- \| \| 2 \| Ao ar livre \| \| 3 \| Ambas \| |
| 103 | afterwk_place6 | Igreja | radio (Matrix)   \| 1 \| Num lugar fechado \| \| --- \| --- \| \| 2 \| Ao ar livre \| \| 3 \| Ambas \| |
| 104 | afterwk_place7 | Shoping | radio (Matrix)   \| 1 \| Num lugar fechado \| \| --- \| --- \| \| 2 \| Ao ar livre \| \| 3 \| Ambas \| |
| 105 | afterwk_place8 | Mercado | radio (Matrix)   \| 1 \| Num lugar fechado \| \| --- \| --- \| \| 2 \| Ao ar livre \| \| 3 \| Ambas \| |
| 106 | afterwk_place9 | Reunião/Encontro com lider comunitário/casa do secretário do bairro | radio (Matrix)   \| 1 \| Num lugar fechado \| \| --- \| --- \| \| 2 \| Ao ar livre \| \| 3 \| Ambas \| |
| 107 | afterwk_place10 | Funeral | radio (Matrix)   \| 1 \| Num lugar fechado \| \| --- \| --- \| \| 2 \| Ao ar livre \| \| 3 \| Ambas \| |
| 108 | afterwk_place11 | Hospital | radio (Matrix)   \| 1 \| Num lugar fechado \| \| --- \| --- \| \| 2 \| Ao ar livre \| \| 3 \| Ambas \| |
| 109 | afterwk_place12 | Unidade Sanitaria | radio (Matrix)   \| 1 \| Num lugar fechado \| \| --- \| --- \| \| 2 \| Ao ar livre \| \| 3 \| Ambas \| |
| 110 | afterwk_place13 | Passear | radio (Matrix)   \| 1 \| Num lugar fechado \| \| --- \| --- \| \| 2 \| Ao ar livre \| \| 3 \| Ambas \| |
| 111 | afterwk_place14 | Outro, especificar | radio (Matrix)   \| 1 \| Num lugar fechado \| \| --- \| --- \| \| 2 \| Ao ar livre \| \| 3 \| Ambas \| |
| 112 | afterwk_time | 43. Qual actividade levou mais tempo? | radio   \| 1 \| Trabalhando \| \| --- \| --- \| \| 2 \| Na machamba \| \| 3 \| Na casa de parentes ou vizinhos (conversando, cozinhando, outros..) \| \| 4 \| Barraca, discoteca, clube \| \| 5 \| Restaurante \| \| 6 \| Igreja \| \| 7 \| Shoping \| \| 8 \| Mercado \| \| 9 \| Reunião/Encontro com lider comunitário/casa do secretário do bairro \| \| 10 \| Funeral \| \| 11 \| Hospital \| \| 12 \| Unidade Sanitaria \| \| 13 \| Passear \| \| 14 \| Outro, especificar \| |
| 113 | afterwk_prev | 44. Você ou a criança usaram algum produto para prevenir picadas de mosquitos DURANTE O DESEMPENHO DE ESSAS ACTIVIDADES? | checkbox   \| 1 \| afterwk_prev___1 \| Spray com insecticida (Baygon) \| \| --- \| --- \| --- \| \| 2 \| afterwk_prev___2 \| Carvão anti-mosquito (Dragão) \| \| 3 \| afterwk_prev___3 \| Repelente \| \| 4 \| afterwk_prev___4 \| Fumo (sulé) \| \| 5 \| afterwk_prev___5 \| Roupa \| \| 6 \| afterwk_prev___6 \| Rede Mosquiteira \| \| 7 \| afterwk_prev___7 \| Nenhum \| \| 8 \| afterwk_prev___8 \| Outros, especificar \| |
| 114 | oth_afterwk_prev | 44.1. Outros, especificar | text |
| 115 | weekend_morning | De manha, voçe faz actividades distintas no final de semana do que durante a semana? | radio   \| 1 \| Sim \| \| --- \| --- \| \| 2 \| Nao \| |
| 116 | reason_nosleep | 45. Porquê não dormiu? | radio   \| 1 \| Estive a trabalhar \| \| --- \| --- \| \| 2 \| Fazia muito calor/frio e não consegui dormir \| \| 3 \| Estava doente \| \| 4 \| Um membro de agregado ficou doente e acompanhei \| \| 5 \| Dando parto \| \| 6 \| Um membro do agregado esteve dando parto e pediu para acompanhar \| \| 7 \| Tenho bebê que tinha que comer ou estava a chorar \| \| 8 \| Estava em casa de amigos ou parentes \| \| 9 \| Viajando \| \| 10 \| Estava na vigília da igreja; \| \| 11 \| Outros, (especifique) \| |
| 117 | oth_reason_nosleep | 45.1. Outros, especificar | text |
| 118 | time_yard | 46. Voce esteve algum tempo no quintal? | radio   \| 1 \| Sim \| \| --- \| --- \| \| 2 \| Não \| \| 3 \| Não sei \| |
| 119 | nosleep_act | 47. Se sim, quais foram as actividades? | checkbox   \| 1 \| nosleep_act___1 \| Cânticos diversos \| \| --- \| --- \| --- \| \| 2 \| nosleep_act___2 \| Somente sentado \| \| 3 \| nosleep_act___3 \| Limpeza \| \| 4 \| nosleep_act___4 \| Cozinhar \| \| 5 \| nosleep_act___5 \| Banho \| \| 6 \| nosleep_act___6 \| Jogos \| \| 7 \| nosleep_act___7 \| Exercícios físicos/desportos \| \| 8 \| nosleep_act___8 \| Socializar (conversar, beber) \| \| 9 \| nosleep_act___9 \| Ver televisão \| \| 10 \| nosleep_act___10 \| Ouvir rádio \| \| 11 \| nosleep_act___11 \| Alimentar ou dar banho a criança/bebe \| \| 12 \| nosleep_act___12 \| Estudar \| \| 13 \| nosleep_act___13 \| Trabalhar \| \| 14 \| nosleep_act___14 \| Outros, especificar \| |
| 120 | oth_nosleep_act | 47.1. Outro, especificar | text |
| 121 | nosleep_place1 | Section Header: *48. Indica para cada actividade seleccionada se foi realizada num local fechado e/ou aberto (ambas podem ser possíveis).*  Cânticos diversos | radio (Matrix)   \| 1 \| Num lugar fechado \| \| --- \| --- \| \| 2 \| Ao ar livre \| \| 3 \| Ambas \| |
| 122 | nosleep_place2 | Somente sentado | radio (Matrix)   \| 1 \| Num lugar fechado \| \| --- \| --- \| \| 2 \| Ao ar livre \| \| 3 \| Ambas \| |
| 123 | nosleep_place3 | Limpeza | radio (Matrix)   \| 1 \| Num lugar fechado \| \| --- \| --- \| \| 2 \| Ao ar livre \| \| 3 \| Ambas \| |
| 124 | nosleep_place4 | Cozinhar | radio (Matrix)   \| 1 \| Num lugar fechado \| \| --- \| --- \| \| 2 \| Ao ar livre \| \| 3 \| Ambas \| |
| 125 | nosleep_place5 | Banho | radio (Matrix)   \| 1 \| Num lugar fechado \| \| --- \| --- \| \| 2 \| Ao ar livre \| \| 3 \| Ambas \| |
| 126 | nosleep_place6 | Jogos | radio (Matrix)   \| 1 \| Num lugar fechado \| \| --- \| --- \| \| 2 \| Ao ar livre \| \| 3 \| Ambas \| |
| 127 | nosleep_place7 | Exercícios físicos/desportos | radio (Matrix)   \| 1 \| Num lugar fechado \| \| --- \| --- \| \| 2 \| Ao ar livre \| \| 3 \| Ambas \| |
| 128 | nosleep_place8 | Socializar (conversar, beber) | radio (Matrix)   \| 1 \| Num lugar fechado \| \| --- \| --- \| \| 2 \| Ao ar livre \| \| 3 \| Ambas \| |
| 129 | nosleep_place9 | Ver televisão | radio (Matrix)   \| 1 \| Num lugar fechado \| \| --- \| --- \| \| 2 \| Ao ar livre \| \| 3 \| Ambas \| |
| 130 | nosleep_place10 | Ouvir rádio | radio (Matrix)   \| 1 \| Num lugar fechado \| \| --- \| --- \| \| 2 \| Ao ar livre \| \| 3 \| Ambas \| |
| 131 | nosleep_place11 | Estudar | radio (Matrix)   \| 1 \| Num lugar fechado \| \| --- \| --- \| \| 2 \| Ao ar livre \| \| 3 \| Ambas \| |
| 132 | nosleep_place12 | Trabalhar | radio (Matrix)   \| 1 \| Num lugar fechado \| \| --- \| --- \| \| 2 \| Ao ar livre \| \| 3 \| Ambas \| |
| 133 | nosleep_place13 | Outros, especificar | radio (Matrix)   \| 1 \| Num lugar fechado \| \| --- \| --- \| \| 2 \| Ao ar livre \| \| 3 \| Ambas \| |
| 134 | nosleep_time | 49. Qual actividade levou mas tempo | radio   \| 1 \| Cânticos diversos \| \| --- \| --- \| \| 2 \| Somente sentado \| \| 3 \| Limpeza \| \| 4 \| Cozinhar \| \| 5 \| Banho \| \| 6 \| Jogos \| \| 7 \| Exercícios físicos/desportos \| \| 8 \| Socializar (conversar, beber) \| \| 9 \| Ver televisão \| \| 10 \| Ouvir rádio \| \| 11 \| Alimentar ou dar banho a criança/bebe \| \| 12 \| Estudar \| \| 13 \| Trabalhar \| \| 14 \| Outros, especificar \| |
| 135 | nosleep_prev | 50. Você ou a criança usaram algum produto para prevenir picadas de mosquitos DURANTE O DESEMPENHO DE ESSAS ACTIVIDADES? | checkbox   \| 1 \| nosleep_prev___1 \| Spray com insecticida (Baygon) \| \| --- \| --- \| --- \| \| 2 \| nosleep_prev___2 \| Carvão anti-mosquito (Dragão) \| \| 3 \| nosleep_prev___3 \| Repelente \| \| 4 \| nosleep_prev___4 \| Fumo (sulé) \| \| 5 \| nosleep_prev___5 \| Roupa \| \| 6 \| nosleep_prev___6 \| Rede Mosquiteira \| \| 7 \| nosleep_prev___7 \| Nenhum \| \| 8 \| nosleep_prev___8 \| Outros, especificar \| |
| 136 | oth_nosleep_prev | 50.1. Outros, especificar | text |
| 137 | nosleep_yard | 51. Voce ou a criança estiveram algum tempo fora do quintal? | radio   \| 1 \| Sim \| \| --- \| --- \| \| 2 \| Não \| \| 3 \| Não sei \| |
| 138 | location_nosleep | 52. Onde você esteve? | checkbox   \| 1 \| location_nosleep___1 \| Trabalhando \| \| --- \| --- \| --- \| \| 2 \| location_nosleep___2 \| Na machamba \| \| 3 \| location_nosleep___3 \| Na casa de parentes ou vizinhos (conversando, cozinhando, outros..) \| \| 4 \| location_nosleep___4 \| Barraca, discoteca, clube \| \| 5 \| location_nosleep___5 \| Restaurante \| \| 6 \| location_nosleep___6 \| Igreja \| \| 7 \| location_nosleep___7 \| Shoping \| \| 8 \| location_nosleep___8 \| Mercado \| \| 9 \| location_nosleep___9 \| Reunião/Encontro com lider comunitário/casa do secretário do bairro \| \| 10 \| location_nosleep___10 \| Funeral \| \| 11 \| location_nosleep___11 \| Hospital \| \| 12 \| location_nosleep___12 \| Unidade Sanitaria \| \| 13 \| location_nosleep___13 \| Passear \| \| 14 \| location_nosleep___14 \| Outro, especificar \| |
| 139 | oth_location_nosleep | 52.1. Outro, especificar | text |
| 140 | othslp_place1 | Section Header: *53. Indica para cada actividade seleccionada se foi realizada num local fechado e/ou aberto (ambas podem ser possíveis).*  Trabalhando | radio (Matrix)   \| 1 \| Num lugar fechado \| \| --- \| --- \| \| 2 \| Ao ar livre \| \| 3 \| Ambas \| |
| 141 | othslp_place2 | Na machamba | radio (Matrix)   \| 1 \| Num lugar fechado \| \| --- \| --- \| \| 2 \| Ao ar livre \| \| 3 \| Ambas \| |
| 142 | othslp_place3 | Na casa de parentes ou vizinhos (conversando, cozinhando, outros..) | radio (Matrix)   \| 1 \| Num lugar fechado \| \| --- \| --- \| \| 2 \| Ao ar livre \| \| 3 \| Ambas \| |
| 143 | othslp_place4 | Barraca, discoteca, clube | radio (Matrix)   \| 1 \| Num lugar fechado \| \| --- \| --- \| \| 2 \| Ao ar livre \| \| 3 \| Ambas \| |
| 144 | othslp_place5 | Restaurante | radio (Matrix)   \| 1 \| Num lugar fechado \| \| --- \| --- \| \| 2 \| Ao ar livre \| \| 3 \| Ambas \| |
| 145 | othslp_place6 | Igreja | radio (Matrix)   \| 1 \| Num lugar fechado \| \| --- \| --- \| \| 2 \| Ao ar livre \| \| 3 \| Ambas \| |
| 146 | othslp_place7 | Shoping | radio (Matrix)   \| 1 \| Num lugar fechado \| \| --- \| --- \| \| 2 \| Ao ar livre \| \| 3 \| Ambas \| |
| 147 | othslp_place8 | Mercado | radio (Matrix)   \| 1 \| Num lugar fechado \| \| --- \| --- \| \| 2 \| Ao ar livre \| \| 3 \| Ambas \| |
| 148 | othslp_place9 | Reunião/Encontro com lider comunitário/casa do secretário do bairro | radio (Matrix)   \| 1 \| Num lugar fechado \| \| --- \| --- \| \| 2 \| Ao ar livre \| \| 3 \| Ambas \| |
| 149 | othslp_place10 | Funeral | radio (Matrix)   \| 1 \| Num lugar fechado \| \| --- \| --- \| \| 2 \| Ao ar livre \| \| 3 \| Ambas \| |
| 150 | othslp_place11 | Hospital | radio (Matrix)   \| 1 \| Num lugar fechado \| \| --- \| --- \| \| 2 \| Ao ar livre \| \| 3 \| Ambas \| |
| 151 | othslp_place12 | Unidade Sanitaria | radio (Matrix)   \| 1 \| Num lugar fechado \| \| --- \| --- \| \| 2 \| Ao ar livre \| \| 3 \| Ambas \| |
| 152 | othslp_place13 | Passear | radio (Matrix)   \| 1 \| Num lugar fechado \| \| --- \| --- \| \| 2 \| Ao ar livre \| \| 3 \| Ambas \| |
| 153 | othslp_place14 | Outro, especificar | radio (Matrix)   \| 1 \| Num lugar fechado \| \| --- \| --- \| \| 2 \| Ao ar livre \| \| 3 \| Ambas \| |
| 154 | othnosleep_time | 54. Qual actividade levou mais tempo? | radio   \| 1 \| Trabalhando \| \| --- \| --- \| \| 2 \| Na machamba \| \| 3 \| Na casa de parentes ou vizinhos (conversando, cozinhando, outros..) \| \| 4 \| Barraca, discoteca, clube \| \| 5 \| Restaurante \| \| 6 \| Igreja \| \| 7 \| Shoping \| \| 8 \| Mercado \| \| 9 \| Reunião/Encontro com lider comunitário/casa do secretário do bairro \| \| 10 \| Funeral \| \| 11 \| Hospital \| \| 12 \| Unidade Sanitaria \| \| 13 \| Passear \| \| 14 \| Outro, especificar \| |
| 155 | othnosleep_prev | 55. Você ou a criança usaram algum produto para prevenir picadas de mosquitos DURANTE O DESEMPENHO DE ESSAS ACTIVIDADES? | checkbox   \| 1 \| othnosleep_prev___1 \| Spray com insecticida (Baygon) \| \| --- \| --- \| --- \| \| 2 \| othnosleep_prev___2 \| Carvão anti-mosquito (Dragão) \| \| 3 \| othnosleep_prev___3 \| Repelente \| \| 4 \| othnosleep_prev___4 \| Fumo (sulé) \| \| 5 \| othnosleep_prev___5 \| Roupa \| \| 6 \| othnosleep_prev___6 \| Rede Mosquiteira \| \| 7 \| othnosleep_prev___7 \| Nenhum \| \| 8 \| othnosleep_prev___8 \| Outros, especificar \| |
| 156 | oth_othnosleep_prev | 55.1. Outro, especificar | text |
| 157 | time_bed | 56. A hora que voce entrou em casa a tarde/noite e nao voltou a sair ate ir para cama/esteira  *Formato deve ser XX:XX* | text |
| 158 | time_sleep | 57.A hora que voçe entrou foi a cama para dormir  *Formato deve ser XX:XX* | text, Required |
| 159 | time_wakeup | 58. A hora que voçe saiu da cama de manha?  *Formato deve ser XX:XX* | text, Required |
| 160 | time_tasks | 59. A hora que voce saiu de casa de manha para começar as tarefas do dia  *Formato deve ser XX:XX* | text, Required |
| 161 | weekend_sleep | 60. Voçê vai a cama mas tarde durante o final de semana? | radio   \| 1 \| Sim \| \| --- \| --- \| \| 2 \| Nao \| |
| 162 | weekend_wakeup | 61. Voçê acorda antes no final de semana? | radio   \| 1 \| Sim \| \| --- \| --- \| \| 2 \| Nao \| |
| 163 | weekend_hours | 62. Em relaçao as horas de sonho durante o final de semana, voçe acha que: | radio   \| 1 \| Durmo mais horas \| \| --- \| --- \| \| 2 \| Durmo menos horas \| \| 3 \| Durmo a mesma quantidade de horas \| |
| 164 | consent_nr | 60. Qual é o número do consentimento informado? | text, Required |
| 165 | inqueridor | 61. Numero do inqueridor | text, Required |
| 166 | questionario_de_comportamento_complete | Section Header: *Form Status*  Complete? | dropdown   \| 0 \| Incomplete \| \| --- \| --- \| \| 1 \| Unverified \| \| 2 \| Complete \| |

*Translation into English*

|  | **Variable / Field Name** | **Field Label**  ***Field Note*** | **Field Attributes (Field Type, Validation, Choices, Calculations, etc.)** |
| --- | --- | --- | --- |
| 1 | uri | URI | text |
| 2 | geopoint_lat | 2.Coordinates_latitude | text |
| 3 | geopoint_lng | 2.Coordinates_longitude | text |
| 4 | geopoint_acc | 2.Coordinates_precisao | text |
| 5 | geopoint_alt | 2.Coordinates_altitude | text |
| 6 | start_date | Date | text (date_dmy) |
| 7 | posto | 3. Administrative division | text |
| 8 | localidade | 3.1. Locality | text |
| 9 | perm_id | 4. ID of the interviewee | text |
| 10 | name | 5. Name of the interviewee | text, Required |
| 11 | p_mainlist | 6. The interviewee is present in the main list of randomly seleted participants | radio   \| 1 \| Yes \| \| --- \| --- \| \| 2 \| No \| |
| 12 | p_present | 7. The interviewee is present? | radio, mandatory   \| 1 \| Yes \| \| --- \| --- \| \| 2 \| No \| |
| 13 | p_reason_present | 8. If not, why not? | radio   \| 1 \| The person emigrated \| \| --- \| --- \| \| 2 \| The person was not at home when the interview was conducted \| \| 3 \| Thy rejected participation \| \| 4 \| The person died \| \| 5 \| There was never a person with that name in the house \| \| 6 \| The person does not have the indicated age \| \| 7 \| The person is not at home, he/she is working outside \| \| 8 \| The ID does not belong to this person \| |
| 14 | age | 9. What is the age of the interviewee? | text (integer) |
| 15 | related_qes | 10. The responses are related to? | radio   \| 1 \| Child 5-11 years old \| \| --- \| --- \| \| 2 \| Child 12-17 years old \| \| 3 \| Adult, she/he has to be the interviewee, \| |
| 16 | p_work | 11. Do you work at night until sunrise (it can also be half time)? | radio   \| 1 \| Yes, formal employment \| \| --- \| --- \| \| 2 \| Yes, self-employed \| \| 3 \| No \| \| 4 \| I do not want to disclose it \| |
| 17 | work_type | 12. Please, specify type of work | text |
| 18 | nigth_work | 13. If yes, do you always work at night? | radio   \| 1 \| Yes \| \| --- \| --- \| \| 2 \| No, I can choose between day or night swifts \| \| 3 \| Only yesterday \| \| 4 \| Others (specify) \| |
| 19 | oth_nigth_work | 13.1. Others (specify) | text |
| 20 | freq_nigth_work | 14. How many days a week do you want at night? | radio   \| 1 \| 1 day \| \| --- \| --- \| \| 2 \| 2 days \| \| 3 \| 3 days \| \| 4 \| 4 days \| \| 5 \| 5 days \| \| 6 \| 6 days \| \| 7 \| 7 days \| \| 8 \| 0 days \| \| 9 \| I don’t know \| |
| 21 | p_respondent | 15. Who is responding to these questions? | radio   \| 1 \| Father/mother \| \| --- \| --- \| \| 2 \| Brother/Sister \| \| 3 \| Grandfather/grandmother \| \| 4 \| The child that has to be interviewed \| \| 5 \| The guardian-caretaker of the child \| |
| 22 | rain | 16. Did it rain last night? | radio   \| 1 \| Yes \| \| --- \| --- \| \| 2 \| No \| \| 3 \| I don’t know \| |
| 23 | change_plans | 17. If yes, did you change your plans for the day? | radio   \| 1 \| Yes \| \| --- \| --- \| \| 2 \| No \| \| 3 \| I don’t know \| |
| 24 | place_sleep | 18. Where did you sleep last night? | radio   \| 1 \| Inside of the house \| \| --- \| --- \| \| 2 \| Outsidee of the house \| \| 3 \| I slept at the yard \| \| 4 \| I didn’t sleep \| |
| 25 | sleep_bednet | 19. Did you, or the kid, slept under the net? | radio   \| 1 \| Yes \| \| --- \| --- \| \| 2 \| No \| \| 3 \| Don’t want to respond \| |
| 26 | yard_before | 20. At night, before going to bed, did you or the kid, were at your compound for some time? | radio   \| 1 \| Yes \| \| --- \| --- \| \| 2 \| No \| \| 3 \| I don’t know \| |
| 27 | actitivities | 21. What activities did you do? | checkbox   \| 1 \| actitivities___1 \| Singing \| \| --- \| --- \| --- \| \| 2 \| actitivities___2 \| I was sitting \| \| 3 \| actitivities___3 \| Cleaning \| \| 4 \| actitivities___4 \| Cooking \| \| 5 \| actitivities___5 \| Taking a bath/shower \| \| 6 \| actitivities___6 \| Playing \| \| 7 \| actitivities___7 \| Physical exercise/sports \| \| 8 \| actitivities___8 \| Socializing (taking, drinking) \| \| 9 \| actitivities___9 \| Watching tv \| \| 10 \| actitivities___10 \| Listening to the radio \| \| 11 \| actitivities___11 \| Feeding or bathing a kid/baby \| \| 12 \| actitivities___12 \| Studying \| \| 13 \| actitivities___13 \| Working \| \| 14 \| actitivities___14 \| Others, specify \| |
| 28 | oth_actitivities | Others, specify | text |
| 29 | act_place1 | Section Header: *22. Indicate, for each activity, if the activity was conducted indoors or outdoors or in both places.*  Singing | radio (Matrix)   \| 1 \| Indoors \| \| --- \| --- \| \| 2 \| Outdoors \| \| 3 \| Both \| |
| 30 | act_place2 | I was sitting | radio (Matrix)   \| 1 \| Indoors \| \| --- \| --- \| \| 2 \| Outdoors \| \| 3 \| Both \| |
| 31 | act_place3 | Cleaning | radio (Matrix)   \| 1 \| Indoors \| \| --- \| --- \| \| 2 \| Outdoors \| \| 3 \| Both \| |
| 32 | act_place4 | Cooking | radio (Matrix)   \| 1 \| Indoors \| \| --- \| --- \| \| 2 \| Outdoors \| \| 3 \| Both \| |
| 33 | act_place5 | Taking a bath/shower | radio (Matrix)   \| 1 \| Indoors \| \| --- \| --- \| \| 2 \| Outdoors \| \| 3 \| Both \| |
| 34 | act_place6 | Playing | radio (radio (Matrix)   \| 1 \| Indoors \| \| --- \| --- \| \| 2 \| Outdoors \| \| 3 \| Both \| |
| 35 | act_place7 | Physical exercise/sports | radio (Matrix)   \| 1 \| Indoors \| \| --- \| --- \| \| 2 \| Outdoors \| \| 3 \| Both \| |
| 36 | act_place8 | Socializing (taking, drinking) | radio (Matrix)   \| 1 \| Indoors \| \| --- \| --- \| \| 2 \| Outdoors \| \| 3 \| Both \| |
| 37 | act_place9 | Watching tv | radio (Matrix)   \| 1 \| Indoors \| \| --- \| --- \| \| 2 \| Outdoors \| \| 3 \| Both \| |
| 38 | act_place10 | Listening to the radio | radio (Matrix)   \| 1 \| Indoors \| \| --- \| --- \| \| 2 \| Outdoors \| \| 3 \| Both \| |
| 39 | act_place11 | Feeding or bathing a kid/baby | radio (Matrix)   \| 1 \| Indoors \| \| --- \| --- \| \| 2 \| Outdoors \| \| 3 \| Both \| |
| 40 | act_place12 | Studying | radio (Matrix)   \| 1 \| Indoors \| \| --- \| --- \| \| 2 \| Outdoors \| \| 3 \| Both \| |
| 41 | act_place13 | Working | radio (Matrix)   \| 1 \| Indoors \| \| --- \| --- \| \| 2 \| Outdoors \| \| 3 \| Both \| |
| 42 | act_place14 | Others (different from the aforementioned activities) | radio (Matrix)   \| 1 \| Indoors \| \| --- \| --- \| \| 2 \| Outdoors \| \| 3 \| Both \| |
| 43 | time_act | 23. Which activitiy took longer? | radio   \| 1 \| Singing \| \| --- \| --- \| \| 2 \| I was sitting \| \| 3 \| Cleaning \| \| 4 \| Cooking \| \| 5 \| Taking a bath/shower \| \| 6 \| Playing \| \| 7 \| Physical exercise/sports \| \| 8 \| Socializing (taking, drinking) \| \| 9 \| Watching tv \| \| 10 \| Listening to the radio \| \| 11 \| Feeding or bathing a kid/baby \| \| 12 \| Studying \| \| 13 \| Working \| \| 14 \| Others, specify \| |
| 44 | prevent_mosquitos | 24. Did you, or the kid, use any product to prevent mosquito bites (during the exercise of the activities)? | checkbox   \| 1 \| prevent_mosquitos___1 \| Spray with insecticide (Baygon) \| \| --- \| --- \| --- \| \| 2 \| prevent_mosquitos___2 \| Charcoal anti mosquitoes (Dragão) \| \| 3 \| prevent_mosquitos___3 \| Repellent \| \| 4 \| prevent_mosquitos___4 \| Smoke (sulé) \| \| 5 \| prevent_mosquitos___5 \| Clothes \| \| 6 \| prevent_mosquitos___6 \| Mosquito net \| \| 7 \| prevent_mosquitos___7 \| None \| \| 8 \| prevent_mosquitos___8 \| Other, specify \| |
| 45 | oth_prevent_mosquitos | 24.1. Others, specify | text |
| 46 | out_yard | 25. At night, before going to bed, did you or the kid spend some time at your yard? | radio   \| 1 \| Yes \| \| --- \| --- \| \| 2 \| No \| \| 3 \| I don’t know \| |
| 47 | where_yard | 26. What were you doing? | checkbox   \| 1 \| where_yard___1 \| Working \| \| --- \| --- \| --- \| \| 2 \| where_yard___2 \| At the farm \| \| 3 \| where_yard___3 \| In the house of relatives or neighbors (talking, cooking, others.) \| \| 4 \| where_yard___4 \| Bar, disco, club \| \| 5 \| where_yard___5 \| Restaurant \| \| 6 \| where_yard___6 \| Church \| \| 7 \| where_yard___7 \| Shopping \| \| 8 \| where_yard___8 \| Market \| \| 9 \| where_yard___9 \| Meeting/Gathering with the community leader/At the house of the neighborhood leader \| \| 10 \| where_yard___10 \| Funeral \| \| 11 \| where_yard___11 \| Hospital \| \| 12 \| where_yard___12 \| Health facility \| \| 13 \| where_yard___13 \| Walking \| \| 14 \| where_yard___14 \| Others, specify \| |
| 48 | oth_where_yard | 26.1. Others, specify | text |
| 49 | oth_place1 | Section Header: *27. Indicate, for each activity, if the activity was conducted indoors or outdoors or in both places.*  Working | radio (Matrix)   \| 1 \| Indoors \| \| --- \| --- \| \| 2 \| Outdoors \| \| 3 \| Both \| |
| 50 | oth_place2 | At the farming land | radio (Matrix)   \| 1 \| Indoors \| \| --- \| --- \| \| 2 \| Outdoors \| \| 3 \| Both \| |
| 51 | oth_place3 | In the house of relatives or neighbors (talking, cooking, others.) | radio (Matrix)   \| 1 \| Indoors \| \| --- \| --- \| \| 2 \| Outdoors \| \| 3 \| Both \| |
| 52 | oth_place4 | Bar, disco, club | radio (Matrix)   \| 1 \| Indoors \| \| --- \| --- \| \| 2 \| Outdoors \| \| 3 \| Both \| |
| 53 | oth_place5 | Restaurant | radio (Matrix)   \| 1 \| Indoors \| \| --- \| --- \| \| 2 \| Outdoors \| \| 3 \| Both \| |
| 54 | oth_place6 | Church | radio (Matrix)   \| 1 \| Indoors \| \| --- \| --- \| \| 2 \| Outdoors \| \| 3 \| Both \| |
| 55 | oth_place7 | Shopping | radio (Matrix)   \| 1 \| Indoors \| \| --- \| --- \| \| 2 \| Outdoors \| \| 3 \| Both \| |
| 56 | oth_place8 | Market | radio (Matrix)   \| 1 \| Indoors \| \| --- \| --- \| \| 2 \| Outdoors \| \| 3 \| Both \| |
| 57 | oth_place9 | Meeting/Gathering with the community leader/At the house of the neighborhood leader | radio (Matrix)   \| 1 \| Indoors \| \| --- \| --- \| \| 2 \| Outdoors \| \| 3 \| Both \| |
| 58 | oth_place10 | Funeral | radio (Matrix)   \| 1 \| Indoors \| \| --- \| --- \| \| 2 \| Outdoors \| \| 3 \| Both \| |
| 59 | oth_place11 | Hospital | radio (Matrix)   \| 1 \| Indoors \| \| --- \| --- \| \| 2 \| Outdoors \| \| 3 \| Both \| |
| 60 | oth_place12 | Health facility | radio (Matrix)   \| 1 \| Indoors \| \| --- \| --- \| \| 2 \| Outdoors \| \| 3 \| Both \| |
| 61 | oth_place13 | Walking | radio (Matrix)   \| 1 \| Indoors \| \| --- \| --- \| \| 2 \| Outdoors \| \| 3 \| Both \| |
| 62 | oth_place14 | Other, specify | radio (Matrix)   \| 1 \| Indoors \| \| --- \| --- \| \| 2 \| Outdoors \| \| 3 \| Both \| |
| 63 | spend_time_place | 28. Where did you or the kid spend most of your time? | radio   \| 1 \| Working \| \| --- \| --- \| \| 2 \| At the farm \| \| 3 \| In the house of relatives or neighbors (talking, cooking, others.) \| \| 4 \| Bar, disco, club \| \| 5 \| Restaurant \| \| 6 \| Church \| \| 7 \| Shopping \| \| 8 \| Market \| \| 9 \| Meeting/Gathering with the community leader/At the house of the neighborhood leader \| \| 10 \| Funeral \| \| 11 \| Hospital \| \| 12 \| Health facility \| \| 13 \| Walking \| \| 14 \| Others, specify \| |
| 64 | prev_mosq | 29. Did you, or the kid, use any product to prevent mosquito bites (during the exercise of the activities)? | checkbox, Required   \| 1 \| prev_mosq___1 \| Spray with insecticide (Baygon) \| \| --- \| --- \| --- \| \| 2 \| prev_mosq___2 \| Charcoal anti mosquitoes (Dragão) \| \| 3 \| prev_mosq___3 \| Repellent \| \| 4 \| prev_mosq___4 \| Smoke (sulé) \| \| 5 \| prev_mosq___5 \| Clothes \| \| 6 \| prev_mosq___6 \| Mosquito net \| \| 7 \| prev_mosq___7 \| None \| \| 8 \| prev_mosq___8 \| Other, specify \| |
| 65 | oth_prev_mosq | 29.1. Others, specify | text |
| 66 | weekend_evening | In the evening, do you do different activities during the weekend than during the week? | radio   \| 1 \| Yes \| \| --- \| --- \| \| 2 \| No \| |
| 67 | out_sleep | 30. Did you get up and leave the bed/mat during sleeping time? | radio   \| 1 \| Yes \| \| --- \| --- \| \| 2 \| No \| \| 3 \| I don’t know \| |
| 68 | reason_out_sleep | 31. If yes, why? | checkbox   \| 1 \| reason_out_sleep___1 \| I received an unexpected visit \| \| --- \| --- \| --- \| \| 2 \| reason_out_sleep___2 \| I had to take care of the animals \| \| 3 \| reason_out_sleep___3 \| I heard an noise and went out to check \| \| 4 \| reason_out_sleep___4 \| I went to the toilet \| \| 5 \| reason_out_sleep___5 \| I woke up due to the baby \| \| 6 \| reason_out_sleep___6 \| I don’t know \| \| 7 \| reason_out_sleep___7 \| Others, specify \| |
| 69 | oth_reason_out_sleep | 31.1. Other, specify | text |
| 70 | night_location_toilet | 31.2 Did you leave the house to go to the toilet? | checkbox   \| 1 \| night_location_toilet___1 \| Yes, to my own toilet \| \| --- \| --- \| --- \| \| 2 \| night_location_toilet___2 \| Yes, in the field \| \| 3 \| night_location_toilet___3 \| No, I used the bucket in my room \| |
| 71 | place_wc | 32. Where is the toilet? | radio   \| 1 \| Outside of the house \| \| --- \| --- \| \| 2 \| Inside of the house \| \| 3 \| In another house/at neighbors’ \| \| 4 \| I don’t have a toilet \| \| 5 \| I don’t want to respond \| \| 6 \| On the open air \| |
| 72 | sleep_prod_prev | 33. Did you, or the kid, use any product to prevent mosquito bites (during the exercise of the activities)? | checkbox   \| 1 \| sleep_prod_prev___1 \| Spray with insecticide (Baygon) \| \| --- \| --- \| --- \| \| 2 \| sleep_prod_prev___2 \| Charcoal anti mosquitoes (Dragão) \| \| 3 \| sleep_prod_prev___3 \| Repellent \| \| 4 \| sleep_prod_prev___4 \| Smoke (sulé) \| \| 5 \| sleep_prod_prev___5 \| Clothes \| \| 6 \| sleep_prod_prev___6 \| Mosquito net \| \| 7 \| sleep_prod_prev___7 \| None \| \| 8 \| sleep_prod_prev___8 \| Other, specify \| |
| 73 | oth_sleep_prod_prev | 33.1. Others, specify | text |
| 74 | sun | 34. When you woke up, had the sun risen? | radio   \| 1 \| Yes \| \| --- \| --- \| \| 2 \| No \| |
| 75 | wake_up_yard | 35. After waking up, and before the sun started to rise, did you spend some time within the yard? | radio   \| 1 \| Yes \| \| --- \| --- \| \| 2 \| No \| \| 3 \| I don’t know \| |
| 76 | wake_actv | 36. Which activities did you do? | checkbox   \| 1 \| wake_actv___1 \| Singing \| \| --- \| --- \| --- \| \| 2 \| wake_actv___2 \| I was sitting \| \| 3 \| wake_actv___3 \| Cleaning \| \| 4 \| wake_actv___4 \| Cooking \| \| 5 \| wake_actv___5 \| Taking a bath/shower \| \| 6 \| wake_actv___6 \| Playing \| \| 7 \| wake_actv___7 \| Physical exercise/sports \| \| 8 \| wake_actv___8 \| Socializing (taking, drinking) \| \| 9 \| wake_actv___9 \| Watching tv \| \| 10 \| wake_actv___10 \| Listening to the radio \| \| 11 \| wake_actv___11 \| Feeding or bathing a kid/baby \| \| 12 \| wake_actv___12 \| Studying \| \| 13 \| wake_actv___13 \| Working \| \| 14 \| wake_actv___14 \| Others, specify \| |
| 77 | oth_wake_actv | 36.1. Others, specify | text |
| 78 | wk_place1 | Section Header: *37. Indica para cada actividade seleccionada se foi realizada num local fechado e/ou aberto (ambas podem ser possíveis).*  Singing | radio (Matrix)   \| 1 \| Indoors \| \| --- \| --- \| \| 2 \| Outdoors \| \| 3 \| Both \| |
| 79 | wk_place2 | I was sitting | radio (Matrix)   \| 1 \| Indoors \| \| --- \| --- \| \| 2 \| Outdoors \| \| 3 \| Both \| |
| 80 | wk_place3 | Cleaning | radio (Matrix)   \| 1 \| Indoors \| \| --- \| --- \| \| 2 \| Outdoors \| \| 3 \| Both \| |
| 81 | wk_place4 | Cooking | radio (Matrix)   \| 1 \| Indoors \| \| --- \| --- \| \| 2 \| Outdoors \| \| 3 \| Both \| |
| 82 | wk_place5 | Taking a bath/shower | radio (Matrix)   \| 1 \| Indoors \| \| --- \| --- \| \| 2 \| Outdoors \| \| 3 \| Both \| |
| 83 | wk_place6 | Playing | radio (Matrix)   \| 1 \| Indoors \| \| --- \| --- \| \| 2 \| Outdoors \| \| 3 \| Both \| |
| 84 | wk_place7 | Physical exercise/sports | radio (Matrix)   \| 1 \| Indoors \| \| --- \| --- \| \| 2 \| Outdoors \| \| 3 \| Both \| |
| 85 | wk_place8 | Socializing (taking, drinking) | radio (Matrix)   \| 1 \| Indoors \| \| --- \| --- \| \| 2 \| Outdoors \| \| 3 \| Both \| |
| 86 | wk_place9 | Watching tv | radio (Matrix)   \| 1 \| Indoors \| \| --- \| --- \| \| 2 \| Outdoors \| \| 3 \| Both \| |
| 87 | wk_place10 | Listening to the radio | radio (Matrix)   \| 1 \| Indoors \| \| --- \| --- \| \| 2 \| Outdoors \| \| 3 \| Both \| |
| 88 | wk_place11 | Feed or bathing a kid/baby | radio (Matrix)   \| 1 \| Indoors \| \| --- \| --- \| \| 2 \| Outdoors \| \| 3 \| Both \| |
| 89 | wk_place12 | Studying | radio (Matrix)   \| 1 \| Indoors \| \| --- \| --- \| \| 2 \| Outdoors \| \| 3 \| Both \| |
| 90 | wk_place13 | Working | radio (Matrix)   \| 1 \| Indoors \| \| --- \| --- \| \| 2 \| Outdoors \| \| 3 \| Both \| |
| 91 | wk_place14 | Others (different from the aforementioned activities) | radio (Matrix)   \| 1 \| Indoors \| \| --- \| --- \| \| 2 \| Outdoors \| \| 3 \| Both \| |
| 92 | wake_act_time | 38. Which activitiy took longer? | radio   \| 1 \| Singing \| \| --- \| --- \| \| 2 \| I was sitting \| \| 3 \| Cleaning \| \| 4 \| Cooking \| \| 5 \| Taking a bath/shower \| \| 6 \| Playing \| \| 7 \| Physical exercise/sports \| \| 8 \| Socializing (taking, drinking) \| \| 9 \| Watching tv \| \| 10 \| Listening to the radio \| \| 11 \| Feeding or bathing a kid/baby \| \| 12 \| Studying \| \| 13 \| Working \| \| 14 \| Others, specify \| |
| 93 | wake_prod_prev | 39. Did you, or the kid, use any product to prevent mosquito bites (during the exercise of the activities)? | checkbox   \| 1 \| wake_prod_prev___1 \| Spray with insecticide (Baygon) \| \| --- \| --- \| --- \| \| 2 \| wake_prod_prev___2 \| Charcoal anti mosquitoes (Dragão) \| \| 3 \| wake_prod_prev___3 \| Repellent \| \| 4 \| wake_prod_prev___4 \| Smoke (sulé) \| \| 5 \| wake_prod_prev___5 \| Clothes \| \| 6 \| wake_prod_prev___6 \| Mosquito net \| \| 7 \| wake_prod_prev___7 \| None \| \| 8 \| wake_prod_prev___8 \| Other, specify \| |
| 94 | oth_wake_prod_prev | 39.1. Others, specify | text |
| 95 | out_afterwk | 40. After waking up, you or the kid spend any time outside of the yard? | radio   \| 1 \| Yes \| \| --- \| --- \| \| 2 \| No \| \| 3 \| I don’t know \| |
| 96 | location_afterwk | 41. Where were you? | checkbox   \| 1 \| location_afterwk___1 \| Working \| \| --- \| --- \| --- \| \| 2 \| location_afterwk___2 \| At the farming land \| \| 3 \| location_afterwk___3 \| In the house of relatives or neighbors (talking, cooking, others) \| \| 4 \| location_afterwk___4 \| Bar, disco, club \| \| 5 \| location_afterwk___5 \| Restaurant \| \| 6 \| location_afterwk___6 \| Church \| \| 7 \| location_afterwk___7 \| Shopping \| \| 8 \| location_afterwk___8 \| Market \| \| 9 \| location_afterwk___9 \| Meeting/Gathering with the community leader/At the house of the neighborhood leader \| \| 10 \| location_afterwk___10 \| Funeral \| \| 11 \| location_afterwk___11 \| Hospital \| \| 12 \| location_afterwk___12 \| Health facility \| \| 13 \| location_afterwk___13 \| Walking \| \| 14 \| location_afterwk___14 \| Other, specify \| |
| 97 | oth_location_afterwk | 41.1. Others, specify | text |
| 98 | afterwk_place1 | Section Header: *42. Indica para cada actividade seleccionada se foi realizada num local fechado e/ou aberto (ambas podem ser possíveis).*  Working | radio (Matrix)   \| 1 \| Indoors \| \| --- \| --- \| \| 2 \| Outdoors \| \| 3 \| Both \| |
| 99 | afterwk_place2 | At the farming land | radio (Matrix)   \| 1 \| Indoors \| \| --- \| --- \| \| 2 \| Outdoors \| \| 3 \| Both \| |
| 100 | afterwk_place3 | In the house of relatives or neighbors (talking, cooking, others.) | radio (Matrix)   \| 1 \| Indoors \| \| --- \| --- \| \| 2 \| Outdoors \| \| 3 \| Both \| |
| 101 | afterwk_place4 | Bar, disco, club | radio (Matrix)   \| 1 \| Indoors \| \| --- \| --- \| \| 2 \| Outdoors \| \| 3 \| Both \| |
| 102 | afterwk_place5 | Restaurant | radio (Matrix)   \| 1 \| Indoors \| \| --- \| --- \| \| 2 \| Outdoors \| \| 3 \| Both \| |
| 103 | afterwk_place6 | Church | radio (Matrix)   \| 1 \| Indoors \| \| --- \| --- \| \| 2 \| Outdoors \| \| 3 \| Both \| |
| 104 | afterwk_place7 | Shopping | radio (Matrix)   \| 1 \| Indoors \| \| --- \| --- \| \| 2 \| Outdoors \| \| 3 \| Both \| |
| 105 | afterwk_place8 | Market | radio (Matrix)   \| 1 \| Indoors \| \| --- \| --- \| \| 2 \| Outdoors \| \| 3 \| Both \| |
| 106 | afterwk_place9 | Meeting/Gathering with the community leader/At the house of the neighborhood leader | radio (Matrix)   \| 1 \| Indoors \| \| --- \| --- \| \| 2 \| Outdoors \| \| 3 \| Both \| |
| 107 | afterwk_place10 | Funeral | radio (Matrix)   \| 1 \| Indoors \| \| --- \| --- \| \| 2 \| Outdoors \| \| 3 \| Both \| |
| 108 | afterwk_place11 | Hospital | radio (Matrix)   \| 1 \| Indoors \| \| --- \| --- \| \| 2 \| Outdoors \| \| 3 \| Both \| |
| 109 | afterwk_place12 | Health facility | radio (Matrix)   \| 1 \| Indoors \| \| --- \| --- \| \| 2 \| Outdoors \| \| 3 \| Both \| |
| 110 | afterwk_place13 | Walking | radio (Matrix)   \| 1 \| Indoors \| \| --- \| --- \| \| 2 \| Outdoors \| \| 3 \| Both \| |
| 111 | afterwk_place14 | Other, specify | radio (Matrix)   \| 1 \| Indoors \| \| --- \| --- \| \| 2 \| Outdoors \| \| 3 \| Both \| |
| 112 | afterwk_time | 43. Which activitiy took longer? | radio   \| 1 \| Working \| \| --- \| --- \| \| 2 \| At the farming land \| \| 3 \| In the house of relatives or neighbors (talking, cooking, others) \| \| 4 \| Bar, disco, club \| \| 5 \| Restaurant \| \| 6 \| Church \| \| 7 \| Shopping \| \| 8 \| Market \| \| 9 \| Meeting/Gathering with the community leader/At the house of the neighborhood leader \| \| 10 \| Funeral \| \| 11 \| Hospital \| \| 12 \| Health facility \| \| 13 \| Walking \| \| 14 \| Other, specify \| |
| 113 | afterwk_prev | 44. Did you, or the kid, use any product to prevent mosquito bites (during the exercise of the activities)? | checkbox   \| 1 \| afterwk_prev___1 \| Spray with insecticide (Baygon) \| \| --- \| --- \| --- \| \| 2 \| afterwk_prev___2 \| Charcoal anti mosquitoes (Dragão) \| \| 3 \| afterwk_prev___3 \| Repellent \| \| 4 \| afterwk_prev___4 \| Smoke (sulé) \| \| 5 \| afterwk_prev___5 \| Clothes \| \| 6 \| afterwk_prev___6 \| Mosquito net \| \| 7 \| afterwk_prev___7 \| None \| \| 8 \| afterwk_prev___8 \| Other, specify \| |
| 114 | oth_afterwk_prev | 44.1. Others specify | text |
| 115 | weekend_morning | In the morning, do you do different activities during the weekend than during the week? | radio   \| 1 \| Yes \| \| --- \| --- \| \| 2 \| No \| |
| 116 | reason_nosleep | 45. Why didn’t you sleep? | radio   \| 1 \| I was working \| \| --- \| --- \| \| 2 \| It was too hot/cold and I couldn’t sleep \| \| 3 \| I was sick \| \| 4 \| A member of the household got sick and I accompanied him/her \| \| 5 \| I gave birth \| \| 6 \| A member of the household was giving birth and I was accompanying \| \| 7 \| I have a baby that had to eat or was crying \| \| 8 \| I was at friends’ or neighbors’ \| \| 9 \| Travelling \| \| 10 \| I was in church; \| \| 11 \| Others, (specify) \| |
| 117 | oth_reason_nosleep | 45.1. Others, specify | text |
| 118 | time_yard | 46. Did you spend some time within the yard? | radio   \| 1 \| Yes \| \| --- \| --- \| \| 2 \| No \| \| 3 \| I don’t know \| |
| 119 | nosleep_act | 47 If yes, what activities did you do? | checkbox   \| 1 \| nosleep_act___1 \| Singing \| \| --- \| --- \| --- \| \| 2 \| nosleep_act___2 \| I was sitting \| \| 3 \| nosleep_act___3 \| Cleaning \| \| 4 \| nosleep_act___4 \| Cooking \| \| 5 \| nosleep_act___5 \| Taking a bath/shower \| \| 6 \| nosleep_act___6 \| Playing \| \| 7 \| nosleep_act___7 \| Physical exercise/sports \| \| 8 \| nosleep_act___8 \| Socializing (taking, drinking) \| \| 9 \| nosleep_act___9 \| Watching tv \| \| 10 \| nosleep_act___10 \| Listening to the radio \| \| 11 \| nosleep_act___11 \| Feeding or bathing a kid/baby \| \| 12 \| nosleep_act___12 \| Studying \| \| 13 \| nosleep_act___13 \| Working \| \| 14 \| nosleep_act___14 \| Others, specify \| |
| 120 | oth_nosleep_act | 47.1. Others, specify | text |
| 121 | nosleep_place1 | Section Header: *48. Indica para cada actividade seleccionada se foi realizada num local fechado e/ou aberto (ambas podem ser possíveis).*  Singing | radio (Matrix)   \| 1 \| Indoors \| \| --- \| --- \| \| 2 \| Outdoors \| \| 3 \| Both \| |
| 122 | nosleep_place2 | I was sitting | radio (Matrix)   \| 1 \| Indoors \| \| --- \| --- \| \| 2 \| Outdoors \| \| 3 \| Both \| |
| 123 | nosleep_place3 | Cleaning | radio (Matrix)   \| 1 \| Indoors \| \| --- \| --- \| \| 2 \| Outdoors \| \| 3 \| Both \| |
| 124 | nosleep_place4 | Cooking | radio (Matrix)   \| 1 \| Indoors \| \| --- \| --- \| \| 2 \| Outdoors \| \| 3 \| Both \| |
| 125 | nosleep_place5 | Taking a bath/shower | radio (Matrix)   \| 1 \| Indoors \| \| --- \| --- \| \| 2 \| Outdoors \| \| 3 \| Both \| |
| 126 | nosleep_place6 | Playing | radio (Matrix)   \| 1 \| Indoors \| \| --- \| --- \| \| 2 \| Outdoors \| \| 3 \| Both \| |
| 127 | nosleep_place7 | Physical exercise/sports | radio (Matrix)   \| 1 \| Indoors \| \| --- \| --- \| \| 2 \| Outdoors \| \| 3 \| Both \| |
| 128 | nosleep_place8 | Socializing (taking, drinking) | radio (Matrix)   \| 1 \| Indoors \| \| --- \| --- \| \| 2 \| Outdoors \| \| 3 \| Both \| |
| 129 | nosleep_place9 | Watching tv | radio (Matrix)   \| 1 \| Indoors \| \| --- \| --- \| \| 2 \| Outdoors \| \| 3 \| Both \| |
| 130 | nosleep_place10 | Listening to the radio | radio (Matrix)   \| 1 \| Indoors \| \| --- \| --- \| \| 2 \| Outdoors \| \| 3 \| Both \| |
| 131 | nosleep_place11 | Feed or bathing a kid/baby | radio (Matrix)   \| 1 \| Indoors \| \| --- \| --- \| \| 2 \| Outdoors \| \| 3 \| Both \| |
| 132 | nosleep_place12 | Studying | radio (Matrix)   \| 1 \| Indoors \| \| --- \| --- \| \| 2 \| Outdoors \| \| 3 \| Both \| |
| 133 | nosleep_place13 | Working | radio (Matrix)   \| 1 \| Indoors \| \| --- \| --- \| \| 2 \| Outdoors \| \| 3 \| Both \| |
| 134 | nosleep_time | 49. Which activity took longer? | radio   \| 1 \| Singing \| \| --- \| --- \| \| 2 \| I was sitting \| \| 3 \| Cleaning \| \| 4 \| Cooking \| \| 5 \| Taking a bath/shower \| \| 6 \| Playing \| \| 7 \| Physical exercise/sports \| \| 8 \| Socializing (taking, drinking) \| \| 9 \| Watching tv \| \| 10 \| Listening to the radio \| \| 11 \| Feeding or bathing a kid/baby \| \| 12 \| Studying \| \| 13 \| Working \| \| 14 \| Others, specify \| |
| 135 | nosleep_prev | 50. Did you, or the kid, use any product to prevent mosquito bites (during the exercise of the activities)? | checkbox   \| 1 \| nosleep_prev___1 \| Spray with insecticide (Baygon) \| \| --- \| --- \| --- \| \| 2 \| nosleep_prev___2 \| Charcoal anti mosquitoes (Dragão) \| \| 3 \| nosleep_prev___3 \| Repellent \| \| 4 \| nosleep_prev___4 \| Smoke (sulé) \| \| 5 \| nosleep_prev___5 \| Clothes \| \| 6 \| nosleep_prev___6 \| Mosquito net \| \| 7 \| nosleep_prev___7 \| None \| \| 8 \| nosleep_prev___8 \| Other, specify \| |
| 136 | oth_nosleep_prev | 50.1. Others, specify | text |
| 137 | nosleep_yard | 51. Did you, or the kid, soend some time outside of the yard? | radio   \| 1 \| Yes \| \| --- \| --- \| \| 2 \| No \| \| 3 \| I don’t know \| |
| 138 | location_nosleep | 52. Where were you? | checkbox   \| 1 \| location_nosleep___1 \| Working \| \| --- \| --- \| --- \| \| 2 \| location_nosleep___2 \| At the farming land \| \| 3 \| location_nosleep___3 \| In the house of relatives or neighbors (talking, cooking, others) \| \| 4 \| location_nosleep___4 \| Bar, disco, club \| \| 5 \| location_nosleep___5 \| Restaurant \| \| 6 \| location_nosleep___6 \| Church \| \| 7 \| location_nosleep___7 \| Shopping \| \| 8 \| location_nosleep___8 \| Market \| \| 9 \| location_nosleep___9 \| Meeting/Gathering with the community leader/At the house of the neighborhood leader \| \| 10 \| location_nosleep___10 \| Funeral \| \| 11 \| location_nosleep___11 \| Hospital \| \| 12 \| location_nosleep___12 \| Health facility \| \| 13 \| location_nosleep___13 \| Walking \| \| 14 \| location_nosleep___14 \| Other, specify \| |
| 139 | oth_location_nosleep | 52.1. Others, specify | text |
| 140 | othslp_place1 | Section Header: *53. Indica para cada actividade seleccionada se foi realizada num local fechado e/ou aberto (ambas podem ser possíveis).*  Working | radio (Matrix)   \| 1 \| Indoors \| \| --- \| --- \| \| 2 \| Outdoors \| \| 3 \| Both \| |
| 141 | othslp_place2 | At the farming land | radio (Matrix)   \| 1 \| Indoors \| \| --- \| --- \| \| 2 \| Outdoors \| \| 3 \| Both \| |
| 142 | othslp_place3 | In the house of relatives or neighbors (talking, cooking, others) | radio (Matrix)   \| 1 \| Indoors \| \| --- \| --- \| \| 2 \| Outdoors \| \| 3 \| Both \| |
| 143 | othslp_place4 | Bar, disco, club | radio (Matrix)   \| 1 \| Indoors \| \| --- \| --- \| \| 2 \| Outdoors \| \| 3 \| Both \| |
| 144 | othslp_place5 | Restaurant | radio (Matrix)   \| 1 \| Indoors \| \| --- \| --- \| \| 2 \| Outdoors \| \| 3 \| Both \| |
| 145 | othslp_place6 | Church | radio (Matrix)   \| 1 \| Indoors \| \| --- \| --- \| \| 2 \| Outdoors \| \| 3 \| Both \| |
| 146 | othslp_place7 | Shopping | radio (Matrix)   \| 1 \| Indoors \| \| --- \| --- \| \| 2 \| Outdoors \| \| 3 \| Both \| |
| 147 | othslp_place8 | Market | radio (Matrix)   \| 1 \| Indoors \| \| --- \| --- \| \| 2 \| Outdoors \| \| 3 \| Both \| |
| 148 | othslp_place9 | Meeting/Gathering with the community leader/At the house of the neighborhood leader | radio (Matrix)   \| 1 \| Indoors \| \| --- \| --- \| \| 2 \| Outdoors \| \| 3 \| Both \| |
| 149 | othslp_place10 | Funeral | radio (Matrix)   \| 1 \| Indoors \| \| --- \| --- \| \| 2 \| Outdoors \| \| 3 \| Both \| |
| 150 | othslp_place11 | Hospital | radio (Matrix)   \| 1 \| Indoors \| \| --- \| --- \| \| 2 \| Outdoors \| \| 3 \| Both \| |
| 151 | othslp_place12 | Health facility | radio (Matrix)   \| 1 \| Indoors \| \| --- \| --- \| \| 2 \| Outdoors \| \| 3 \| Both \| |
| 152 | othslp_place13 | Walking | radio (Matrix)   \| 1 \| Indoors \| \| --- \| --- \| \| 2 \| Outdoors \| \| 3 \| Both \| |
| 153 | othslp_place14 | Other, specify | radio (Matrix)   \| 1 \| Indoors \| \| --- \| --- \| \| 2 \| Outdoors \| \| 3 \| Both \| |
| 154 | othnosleep_time | 54. Which activitiy took longer? | radio   \| 1 \| Working \| \| --- \| --- \| \| 2 \| At the farming land \| \| 3 \| In the house of relatives or neighbors (talking, cooking, others) \| \| 4 \| Bar, disco, club \| \| 5 \| Restaurant \| \| 6 \| Church \| \| 7 \| Shopping \| \| 8 \| Market \| \| 9 \| Meeting/Gathering with the community leader/At the house of the neighborhood leader \| \| 10 \| Funeral \| \| 11 \| Hospital \| \| 12 \| Health facility \| \| 13 \| Walking \| \| 14 \| Other, specify \| |
| 155 | othnosleep_prev | 55. Did you, or the kid, use any product to prevent mosquito bites (during the exercise of the activities)? | checkbox   \| 1 \| othnosleep_prev___1 \| Spray with insecticide (Baygon) \| \| --- \| --- \| --- \| \| 2 \| othnosleep_prev___2 \| Charcoal anti mosquitoes (Dragão) \| \| 3 \| othnosleep_prev___3 \| Repellent \| \| 4 \| othnosleep_prev___4 \| Smoke (sulé) \| \| 5 \| othnosleep_prev___5 \| Clothes \| \| 6 \| othnosleep_prev___6 \| Mosquito net \| \| 7 \| othnosleep_prev___7 \| None \| \| 8 \| othnosleep_prev___8 \| Other, specify \| |
| 156 | oth_othnosleep_prev | 55.1. Others, specify | text |
| 157 | time_bed | 56. Time when you entered the house in the evening/night after which you did not go out anymore before you went to bed  *Time format HH:MM* | text |
| 158 | time_sleep | 57.Time when you went to bed  *Time format HH:MM* | text, Required |
| 159 | time_wakeup | 58. Time when you got up  *Time format HH:MM* | text, Required |
| 160 | time_tasks | 59. Time when you left the house in the morning for start your activities  *Time format HH:MM* | text, Required |
| 161 | weekend_sleep | 60. Do you go to bed later during the weekends? | radio   \| 1 \| Yes \| \| --- \| --- \| \| 2 \| No \| |
| 162 | weekend_wakeup | 61. Do you get up earlier during the weekends? | radio   \| 1 \| Yes \| \| --- \| --- \| \| 2 \| No \| |
| 163 | weekend_hours | 62. In relation to sleeping times during the weekends, do you believe that: | radio   \| 1 \| I sleep longer \| \| --- \| --- \| \| 2 \| I sleep less hours \| \| 3 \| I sleep the same amount of time \| |
| 164 | consent_nr | 60. What is the number of the informed consent? | text, Required |
| 165 | inqueridor | 61. Field worker number | text, Required |
| 166 | questionario_de_comportamento_complete | Section Header: *Form Status*  Complete? | dropdown   \| 0 \| Incomplete \| \| --- \| --- \| \| 1 \| Unverified \| \| 2 \| Complete \| |
